# Concise and Scalable Radiosynthesis of (+)-[^18^F]MDL100907 as a Serotonin 5-HT_2A_ Receptor Antagonist for PET

**DOI:** 10.1021/acschemneuro.3c00382

**Published:** 2023-09-25

**Authors:** Lahu N. Chavan, Ronald Voll, Mar M. Sanchez, Jonathon A. Nye, Mark M. Goodman

**Affiliations:** † Department of Radiology and Imaging Science, Emory University School of Medicine, Atlanta, Georgia 30329, United States; ‡ Department of Psychiatry and Behavioral Sciences, Emory National Primate Center, Emory University School of Medicine, Atlanta, Georgia 30322, United States; § Department of Radiology and Imaging Sciences Wesley Woods Health Center, 1841 Clifton Rd. NE, 2nd Floor, Atlanta, Georgia 30329, United States

**Keywords:** 5-HT_2A_, radiofluorination, copper catalysis, Liebeskind−Srogl reaction

## Abstract

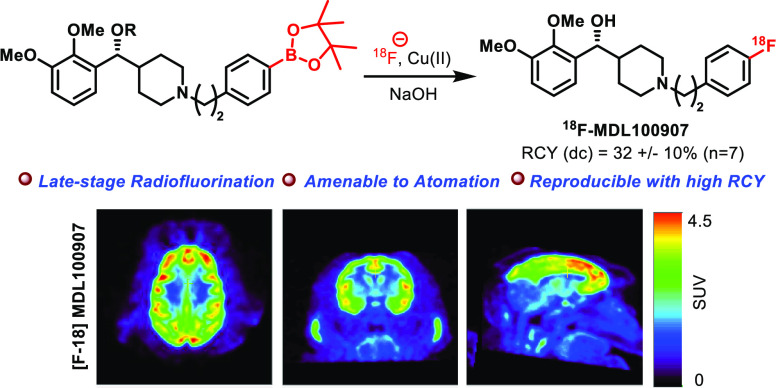

5-Hydroxytryptamine (5-HT_2A_) receptors play an important role in several psychiatric disorders. In order to investigate the serotonin (5-HT) receptor *in vivo*, reliable syntheses are required for positron emission tomography (PET) 5-HT radioligands. Owing to the excellent *in vivo* properties of [^18^F]MDL100907 for PET, there has been great interest to develop a novel synthetic route for [^18^F]MDL100907. Here, we report a highly efficient, scalable, and expedient synthesis for [^18^F]MDL100907. The radiofluorination was performed on a ^18^F-labeling boron pinacol ester precursor, which is synthesized using the Liebeskind–Srogl cross-coupling reaction as a key step. Our method is practically more suitable to employ late-stage Cu-mediated radiofluorination and facilitate the production of the [^18^F]MDL100907 radioligand in excellent decay-corrected RCY of 32 ± 10% (*n* = 7) within 60 min. We prepared [^18^F]MDL100907 in high molar activity (2.1 Ci/μmol) and compared it to [^11^C]MDL100907 in the brain of a nonhuman primate.

## Introduction

**Figure 1:**
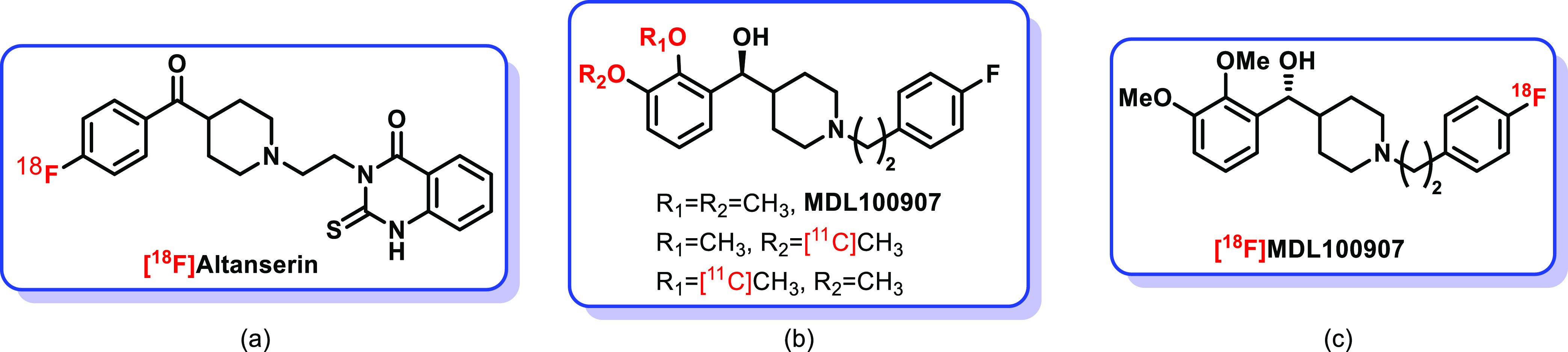
PET-based radioligands.

In the brain, serotonin, or 5-hydroxytryptamine (5-HT), is released from serotonergic fibers that originate in the raphe nuclei and innervate cortical and limbic structures of the medial temporal lobe system.^1−3^ Serotonin receptors are an important neurotransmitter in the central nervous system (CNS) and peripheral tissues, which play a crucial role in the pathophysiology of several neuropsychiatric disorders. There are seven major families of transmembrane receptors (5-HT_1–7_), and one transporter is known to control 5-HT function.^4,5^ Literature findings indicate that serotonergic 5-HT_2A_ receptors, which are widely expressed in cortical and forebrain regions, may be related to or contribute to neuropsychiatric disorders, such as schizophrenia, major depressive disorder, bipolar disorder, and cognitive disturbances associated with Alzheimer’s or Parkinson’s disease.^6−9^ Serotonergic 5-HT_2A_ receptors are of central interest in the quantification of receptor densities and measurement of receptor occupancy in the human brain to understand the biological principles of the mentioned disorders and contribute to the development of appropriate therapies.^10−15^ To quantify 5-HT_2A_ receptors *in vivo*, molecular imaging techniques, such as positron emission tomography (PET) or single-photon emission computed tomography, can be used. There have been large efforts done to develop selective and high-affinity radiolabeled ligands for PET-based visualization of 5-HT_2A_, of which [^11^C]MDL100907 or [^18^F]MDL100907 and [^18^F]altanserin proved to be the most useful (Figure 1).^16−24^

*In vivo* studies of both [^11^C]MDL100907 and [^18^F]altanserin tracers showed high affinity and selectivity for 5-HT_2A_ receptors. However, [^18^F]altanserin metabolizes rapidly and forms at least four metabolites, which penetrate the BBB and hinder the measurement of uptake kinetics in the brain.^25^ Additionally, [^18^F]altanserin shows off-target binding to receptors outside the serotonergic system. On the other hand, MDL100907 undergoes extensive first-pass metabolism, and the major metabolites of MDL100907 do not enter the brain to any large extent.^26−28^ Thus, the [^11^C]MDL100907- and [^18^F]MDL100907-labeled tracers have been synthesized and used in PET imaging. In 1999, Mathis et al. reported the ^11^C-labeling of MDL100907 in two specific positions.^26^ But the major drawback of [^11^C]MDL100907 is its short half-life (20 min), which makes it difficult to measure the binding of ligands during late time points (≥90 min) before the ligand equilibrates between blood and tissue.^29,31^

**Scheme 1:**
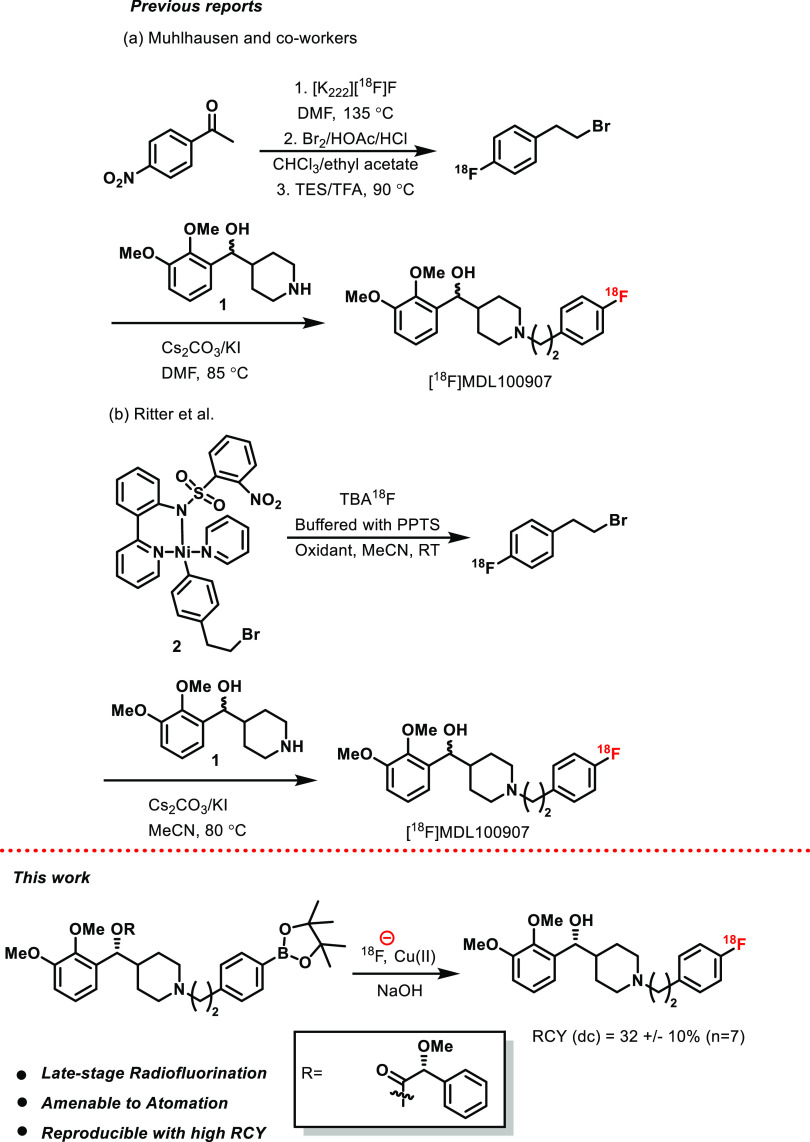
Previous and Present Approaches

Apart from [^11^C]MDL100907, a radioligand [^18^F]MDL100907 with a more suitable half-life has been synthesized by Mühlhausen and co-workers.^30^ However, the complex labeling step involved a sequential four-step reaction sequence with 120 min of reaction time, which ended with a low (1–2%) radiochemical yield (Scheme 1). Recently, Ritter et al. demonstrated the Ni-mediated oxidative fluorination for the synthesis of [^18^F]MDL100907.^31^ The reaction sequence involved in this protocol is a one-pot two-step process. Initially, the precursor **2** was labeled with ^18^F, which was then assembled with an excess of chiral amine **1** to offer desired [^18^F]MDL100907 in a 3% non-decay-corrected yield (Scheme 1).

Here, we report a novel synthetic strategy for producing [^18^F]MDL100907 in high RCY^32^ through a late-stage Cu-mediated radiofluorination reaction. The resulted [^18^F]MDL100907 was evaluated against [^11^C]MDL100907 for reliability to quantify 5-HT_2A_ in the nonhuman primate brain. For this endeavor, we have chosen the classical Liebeskind–Srogl cross-coupling reaction as the key transformation for the scalable synthesis of compound **6**.

## Results and Discussion

**Scheme 2:**
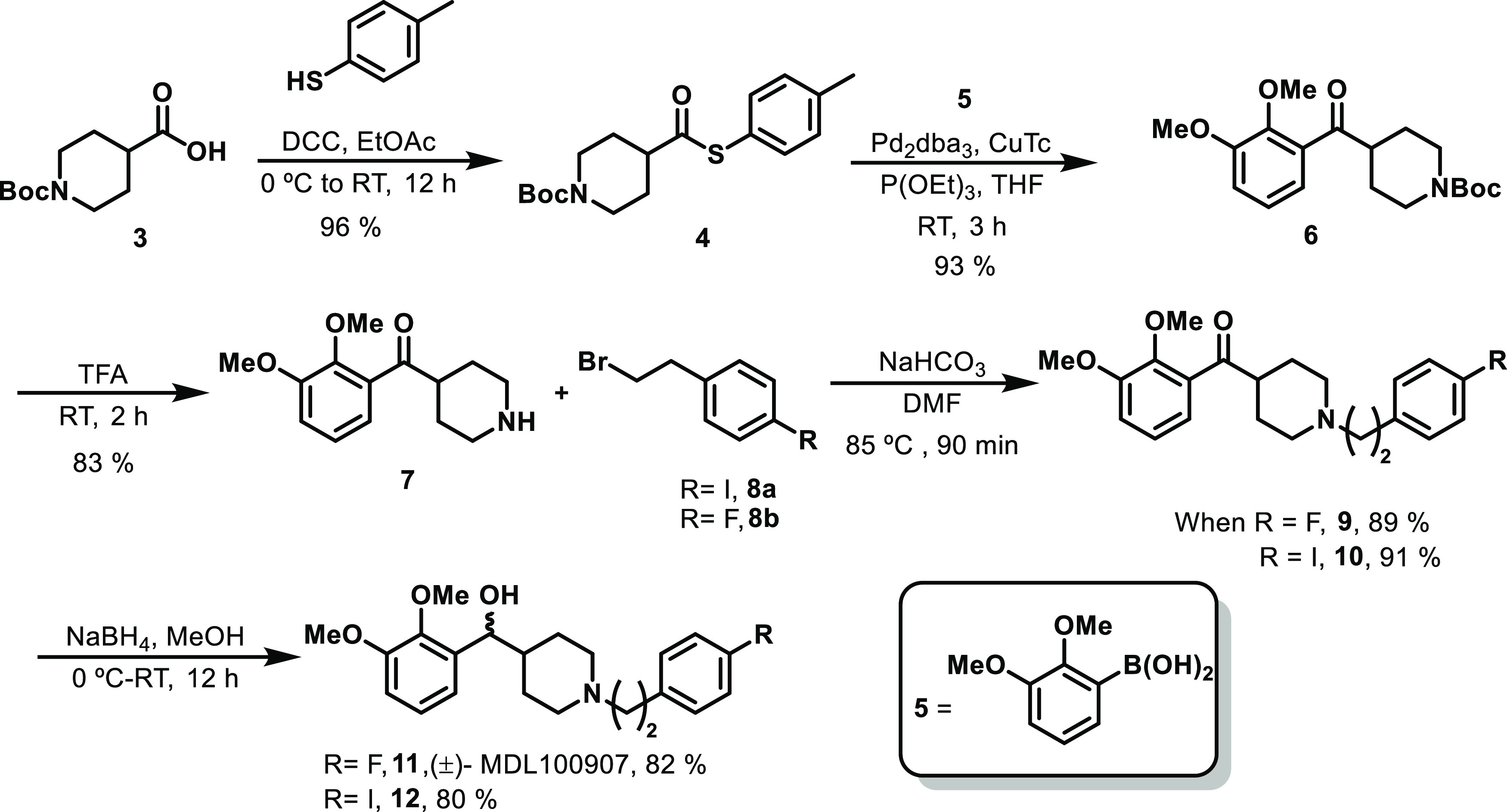
Synthesis of (±) MDL100907 and Iodo-Analogue

Our synthetic strategy of **6** depicted in Scheme 2 relied on a copper-mediated palladium-catalyzed Liebeskind–Srogl cross-coupling reaction of thioester derivative **4** and boronic acid **5** under neutral conditions.^33,34^ In our attempt to synthesize compound **6** in quantitative yield, we have optimized the reaction conditions using different copper complexes (Table 1). In this cross-coupling, copper(I) diphenylphosphinate (CuDPP) was the best Cu(I) carboxylate complex, giving a high yield of ketone product.

**Table 1:**
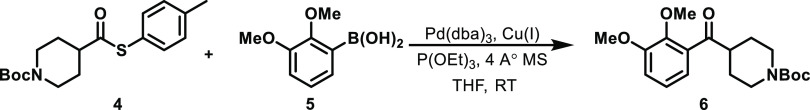
Screening for Optimal Reaction Conditions^a^^,^^b^

entry	Cu(I)	time (h)	yield
1	CuTC	4.5	76
2	CuDPP	4.5	93
3	CuMeSal	4.5	81

a Standard reaction conditions: The reaction was carried out with **4** (1 mmol), **5** (2 mmol), Pd(dba)_3_ (0.025 mmol) Cu(I) (1.5 mmol), and P(OEt)_3_ (0.2 mmol) in solvent (0.1 M).

b Yield of the isolated product.

Briefly, the synthesis commenced with the coupling of commercially available acid **3** with *p*-toluene thiophenol using dicyclohexylcarbodiimide (DCC) at room temperature resulting in corresponding thioester **4** in a 96% yield.^35^ With an appropriate precursor in hand, we next investigated the Liebeskind–Srogl cross-coupling reaction of thioester **4** and boronic acid **5** with various copper reagents, such as copper(I) thiophene-2-carboxylate (CuTC), CuDPP, and copper(I) methylsalicylate (CuMeSal), using a catalytic amount of Pd(dba)_3_ in THF solvent at room temperature (RT). To our delight, CuDPP was found to be the better reagent to yield a key ketone product in 93% yield.

In the next synthetic step, the removal of the piperidine Boc group with TFA produced **7** in 83% yield. The free amine **7** was subsequently coupled with 4-iodophenylethyl bromide **8a** (prepared by the reaction of 4-iodophenylethyl alcohol with PBr_3_ in dichloromethane) and 4-fluorophenylethyl bromide **8b**, which provided **9** and **10** in 91 and 89% yields, respectively. The ketone groups in **9** and **10** were reduced using NaBH_4_ in methanol, which provided racemic (±) MDL100907 (**11**) and iodo-derivative **12** in high yield (Scheme 2).

**Scheme 3:**
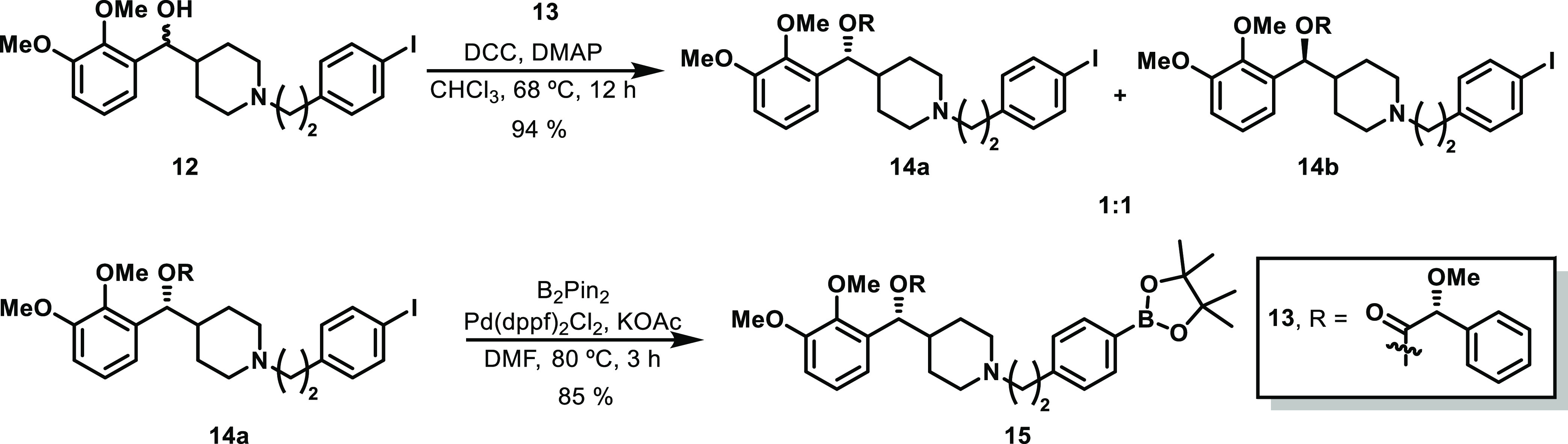
Synthesis of Pinacol Boron Ester Precursor

The racemic alcohol **12** was derivatized with chiral (*s*)-(+)-α-methoxyphenylacetic acid to afford two diastereomers in 93% yield (**14a**, **14b** in 1:1 ratio), which were easily separated by column chromatography. The radiolabeling precursor **15** was synthesized from the required diastereomer **14a** by a Miyaura borylation reaction using bispinacolatodiboron and Pd(dppf)Cl_2_ in 85% yield after purification.^36^ Thus, this multistep procedure proved successful and allowed us to synthesize the desired borylated precursor on a gram scale (Scheme 3).

**Scheme 4:**
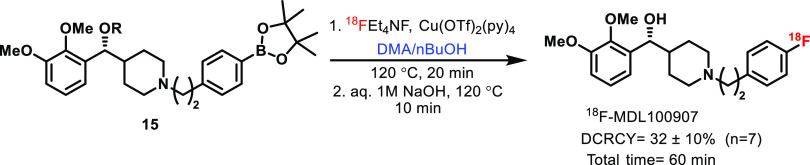
Cu-Mediated Late-Stage Radiolabeling^,^ Reaction conditions: The reaction was carried out with **15** (1.5 mmol), Cu(OTf)_2_(py)_4_ (3.1 mmol), fluoride source, and TEABC (1.5 mg) in DMA/nBuOH (300/100 μL). Decay-corrected radiochemical yields of products isolated after HPLC purification.

Having synthesized borylated precursor **14a**, we next focused on exploring the copper-mediated oxidative ^18^F-fluorination reaction.^37−39^ Typically, the most common catalytic condition used in oxidative ^18^F-fluorination is Cu(OTf)_2_(py)_4_^40−43^ either in DMA or DMF as the solvent at 120 °C temperature for 20 to 30 min to acquire better conversion.^44^ Initially, we attempted direct radiofluorination on **15** using remote semiautomated synthesis in a Siemens Computer Programmable Chemical Unit (CPCU) using DMA at 110 °C, followed by deprotection of the ester group (aq 1 N NaOH, 10 min at 120 °C) furnished [^18^F]MDL100907 in low radiochemical yield, RCY = 1% along with protodeboronation.^45^ This was circumvented by heating the reaction mixture at 120 °C, where [^18^F]MDL100907 was obtained in RCY = 3–6% (*n* = 6). Surprisingly, we did not observe any protodeboronation product during high-performance liquid chromatography (HPLC) purification, but the yield of the reaction was not satisfactory. Anticipating a better yield, we used *n*BuOH as a co-solvent along with DMA; to our delight, the reaction yield was increased substantially, and we obtained [^18^F]MDL100907 in 32 ± 10% (*n* = 7). Eventually, the pinacol ester **15** was radiofluorinated using the Cu(II) catalyst with subsequent deprotection of the ester group using 1 M NaOH as the base, followed by HPLC purification that required only 60 min (Scheme 4).^46^ The enantiomeric purity (ee) of the final product was 92.61 ± 0.64% (*n* = 2) checked after the basic hydrolysis of ester, as shown in Figures S20–S22.

**Figure 2:**
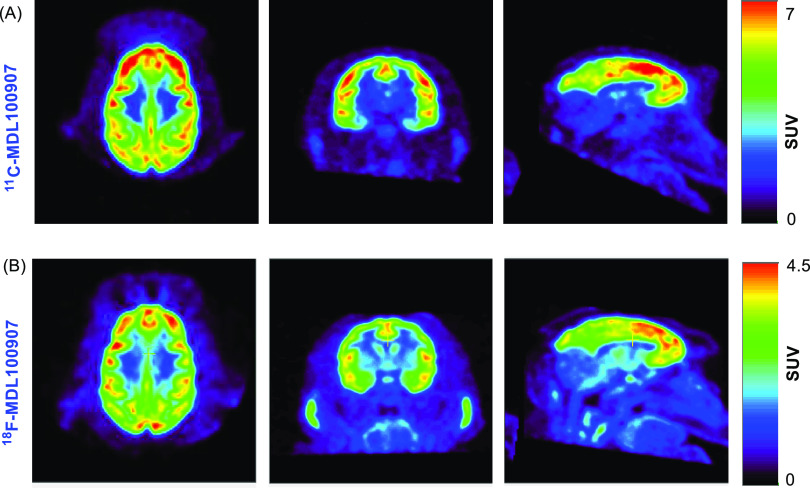
5-HT_2A_ distribution microPET brain uptake.

The microPET images for a 120 min study with [^18^F]MDL100907 in an adult male rhesus monkey and in comparison with [^11^C]MDL100907 for a 90 min study taken in the same male rhesus monkey 3 years earlier are shown in Figure 2. The time–activity curves (TACs) for [^18^F]MDL100907 and [^11^C]MDL100907 are shown in Figures S24 and S25, respectively. As shown in Figures S24 and S25, high uptake of radioactivity is observed in the regions of the brain known to have a high 5-HT_2a_ density.^27^

In conclusion, a flexible and scalable synthetic route to MDL100907 and precursor for radiosynthesis of [^18^F]MDL100907 **15** has been developed using the Liebeskind–Srogl cross-coupling reaction as a key step to introduce the aryl ketone moiety. Our method is practically more suitable to employ late-stage Cu-mediated radiofluorination and facilitate the production of [^18^F]MDL100907 in excellent DCRCY of 32 ± 10% (*n* = 7) within 60 min, 92.61% ee with high molar activity (2.1 Ci/μmol) (see the Supporting Information). As demonstrated by our initial finding, [^18^F]MDL100907 provides a comparable 5-HT_2A_ distribution to [^11^C]MDL100907 as shown in Figure 2. Repetitive scans and analyses with [^18^F]MDL100907 are in progress to demonstrate the robustness of this method for translation to human imaging for clinical neuroscience research.

## Materials and Methods

### General Information

All solvents were purchased from Fisher Scientific or Sigma-Aldrich and dried over 4 Å molecular sieves (8–12 mesh, Sigma-Aldrich). Unless otherwise noted, all commercially available reagents and substrates were used directly as received. Thin-layer chromatography was performed on Merck silica gel plates and visualized by ultraviolet (UV) light and/or potassium permanganate. ^1^H, ^13^C, and ^19^F NMR spectra were recorded on Bruker 300, Varian INOVA 600, INOVA 500, and INOVA 400 spectrometers. Residual solvent resonances were treated as internal reference signals. ^19^F spectra were referenced to either trifluoroacetic acid (−76.55 ppm) or fluorobenzene (−113.15 ppm). IR spectra were recorded on a Nicolet iS10 Fourier-transform infrared (FTIR) spectrometer, and the absorption peaks were reported in cm^–1^. The purification of products was performed via flash chromatography, unless otherwise noted. High-resolution mass spectra were obtained from the Emory University Mass Spec Facility Inc. The [^18^F]fluoride was produced at the Emory University Center for Systems Imaging with an 11 MeV Siemens RDS 111 negative-ion cyclotron (Knoxville, TN) by the ^18^O(p,n) ^18^F reaction using [^18^O]H_2_O (95%). Alumina N SepPaks and HLB Oasis cartridges were purchased from Waters, Inc. (Milford, MA). Radiometric TLC was performed with the same type of silica plates from Whatman and analyzed using a Raytest system (Rita Star, Germany). Isolated radiochemical yields were determined using a dose calibrator (Capintec CRC-712M). Analytical HPLC experiments were performed with a Waters Breeze HPLC system equipped with a Bioscan flowcount radioactivity detector and an inline UV detector set to monitor wavelengths 210, 230, and 254 nm (Astec chirobiotic T column, Sigma-Aldrich part number 12021AST; mobile phase: MeOH). All animal experiments were carried out under humane conditions and were approved by the Institutional Animal Use and Care Committee (IUCAC) and Radiation Safety Committees at Emory University.

#### General Procedure for the Synthesis of *tert*-Butyl 4-((*p*-tolylthio)carbonyl)piperidine-1-carboxylate (**4**)

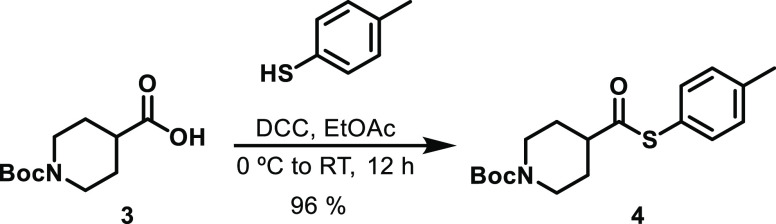



To a stirred solution of Boc-Inp-OH **3** (10 g, 43.6 mmol, 1 equiv) and 4-methylbenzenethiol (8.1 g, 65.5 mmol, 1.5 equiv) in dry ethyl acetate (0.1 M) at 0 °C were added *N*,*N*′-dicyclohexylcarbodiimide (9.8 g, 47.9 mmol, 1.1 equiv) and HOBt (7.7 g, 65.4 mmol, 1.5 equiv) under a N_2_ atmosphere. The reaction was stirred at 0 °C for the first 30 min and then at room temperature overnight. After completion of the reaction, a few drops of 50% acetic acid in ethyl acetate was added and the reaction mixture was filtered through a pad of celite. The filtrate was dried over Na_2_SO_4_, and the solvent was removed under reduced pressure and the crude reaction mixture was purified by column chromatography on silica gel to afford **3** as a white solid (14 g, 96% yield). *R*_f_ = 0.3 (EtOAc/hexane, 7:3); ^1^H NMR (300 MHz, CDCl_3_) δ 7.32–7.14 (m, 4H), 4.09 (d, *J* = 11.1 Hz, 2H), 2.90–2.63 (m, 3H), 2.35 (s, 3H), 1.93 (d, *J* = 10.9 Hz, 2H), 1.79–1.61 (m, 2H), 1.45 (s, 9H). ^13^C NMR (75 MHz, CDCl_3_) δ 199.8, 154.6, 139.7, 135.2–133.7 (m), 130.8–129.1 (m), 123.7, 79.7, 50.0 (d, *J* = 34.5 Hz), 44.1–42.0 (m Hz), 29.6–27.2 (m), 21.3 (d, *J* = 12.0 Hz); high-resolution mass spectrometry (HRMS) (ESI) calcd for C_21_H_14_O_2_ [M + H]^+^: 299.1072; found: 299.1084.

#### General Procedure for the Synthesis of *tert*-Butyl 4-(2,3-dimethoxybenzoyl)piperidine-1-carboxylate (**6**)

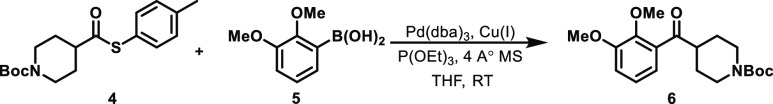



##### General Procedure for the Liebeskind–Srogl Cross-Coupling Reaction

A mixture of *N*-Boc-piperidine-Phe-SPh (12.7 g, 38.0 mmol, 1 equiv), 2, 3-dimethoxylphenylboronic acid (13.8 g, 76.1 mmol, 2.0 equiv), CuDPP (10.8 g, 57.0 mmol, 1.5 equiv), and Pd_2_(dba)_3_ (0.869 g, 0.025 mmol, 0.025 equiv) was placed under an argon atmosphere. THF (110 mL, degassed and dried over 4 Å molecular sieves) and triethylphosphite (1.2 g, 7.6 mmol, 0.2 equiv) were added, and the mixture was stirred at room temperature until the *N*-Boc-piperidine-Phe-SPh ester was consumed (∼3 h). Reaction progress was monitored by TLC. The reaction mixture was diluted with ether (200 mL), washed with aq NaHCO_3_ (200 mL ×2) solution and brine (150 mL), and then dried over Na_2_SO_4_. The drying agent was filtered off through a short plug of silica gel (to aid removal of metal-containing products) and concentrated under vacuum using a rotary evaporator. The crude product was purified on silica gel column chromatography to give the insertion product **6** as a pale oil (12.2 g, 93% yield). *R*_f_ = 0.3 (EtOAc/hexane, 6:4); ^1^H NMR (300 MHz, CDCl_3_) δ 7.13–6.86 (m, 3H), 4.14–3.95 (m, 2H), 3.86 (s, 3H), 3.83 (s, 3H), 3.20 (tt, *J* = 11.0, 3.7 Hz, 1H), 2.79 (t, *J* = 11.5 Hz, 2H), 1.81 (dd, *J* = 13.2, 2.6 Hz, 2H), 1.63–1.44 (m, 3H), 1.41 (s, 9H).; ^13^C NMR (75 MHz, CDCl_3_) δ 206.0, 154.7 (d, *J* = 6.2 Hz), 154.2, 152.7, 151.5 (d, *J* = 9.6 Hz), 146.9, 134.0, 128.2–127.1 (m), 125.0–124.1 (m), 120.5–120.0 (m), 116.2–114.7 (m), 79.5, 61.4 (dd, *J* = 30.0, 14.6 Hz), 57.0–55.4 (m), 48.0, 44.0–42.5 (m), 28.4, 28.3, 27.8.

#### General Procedure for the Synthesis of (2,3-Dimethoxyphenyl)(piperidin-4-yl)methanone (**7**)

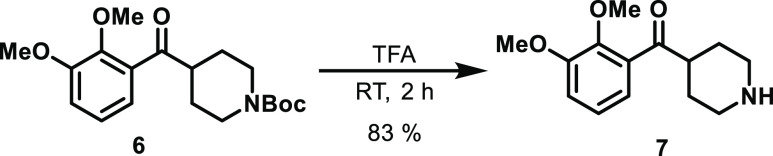



The 4-(2,3-bismethoxy-benzoyl)-1-piperidinecarboxylic acid *t*-butyl ester (10 g, 28.6 mmol) was dissolved carefully and gradually into trifluoroacetic acid (100 mL). The mixture was stirred at room temperature for 2 h. After that, the reaction mixture was diluted with 50 mL of ether and neutralized carefully with NH_4_OH while maintaining cooling in an ice bath. The layers were separated, and the aqueous layer was extracted with ether (3 × 50 mL). The combined organic extracts were washed with water (50 mL), dried over Na_2_SO_4_, filtered the combined organic extracts, and evaporated to afford a crude viscous brown oil (5.9 g, 83% yield). *R*_f_ = 0.2 (20%, CHCl_3_/MeOH = 8:2); ^1^H NMR (75 MHz, CD_3_OD) δ 7.23–7.03 (m, 2H), 6.97–6.91 (m Hz, 1H), 3.89–3.85 (m, 3H), 3.85–3.83 (m, 3H), 3.28–3.15 (m Hz, 1H), 3.11–2.97 (m Hz, 2H), 2.69–2.55 (m Hz, 2H), 1.88–1.75 (m Hz, 2H), 1.62–1.44 (m Hz, 2H). ^13^C NMR (75 MHz, CD_3_OD) δ 206.5, 152.8, 146.9, 133.8, 124.3 (d, *J* = 36.8 Hz), 119.9 (d, *J* = 34.9 Hz), 114.9 (d, *J* = 33.8 Hz), 60.5 (d, *J* = 22.8 Hz), 54.9 (d, *J* = 23.0 Hz), 45.0 (t, *J* = 24.3 Hz), 28.5–27.7 (m).

#### (2,3-Dimethoxyphenyl)(1-(4-fluorophenethyl)piperidin-4-yl)methanone (**9**)

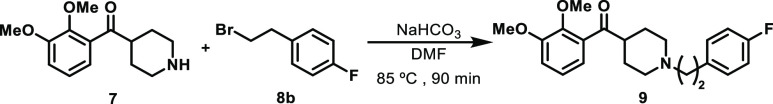



To a stirred suspension of (2,3-dimethoxyphenyl)(piperidin-4-yl)methanone **7** (100 mg, 0.4 mmol, 1 equiv) and NaHCO_3_ (50 mg, 0.6 mmol, 1.5 equiv) in DMF (0.2 mL) was added 1-(2-bromoethyl)-4-iodobenzene (98 mg, 4.8 mmol, 1.2 equiv) under a N_2_ atmosphere, and the reaction mixture was heated at 85 °C for 90 min. After the starting material disappeared, the reaction mixture was cooled to room temperature and water was added. Then, the mixture was extracted with ethyl acetate (3 × 50 mL). The combined organic layers were dried over anhydrous Na_2_SO_4_ and concentrated under reduced pressure. The residue was purified by silica gel chromatography to give **9** as a colorless oil (133 g, 89% yield). *R*_f_ = 0.3 (EtOAc/hexane, 6:4); ^1^H NMR (300 MHz, CDCl_3_) δ 7.17–7.04 (m, 3H), 7.02–6.89 (m, 4H), 3.87 (s, 3H), 3.85 (s, 3H), 3.07 (tt, *J* = 11.0, 3.9 Hz, 1H), 2.96 (dt, *J* = 11.4, 3.2 Hz, 2H), 2.80–2.71 (m, 2H), 2.58–2.48 (m, 2H), 2.10 (td, *J* = 11.4, 2.5 Hz, 2H), 1.96–1.84 (m, 2H), 1.81–1.65 (m, 2H); ^13^C NMR (75 MHz, CDCl_3_) δ 206.4, 162.9, 159.7, 152.7, 146.9, 136.0, 134.3, 130.8–130.0 (m), 124.6 (d, *J* = 39.2 Hz), 120.5 (d, J = 39.1 Hz), 116.2–113.6 (m), 61.6 (d, *J* = 23.8 Hz), 60.7, 55.8 (d, *J* = 23.5 Hz), 53.1 (d, *J* = 31.4 Hz), 47.9 (d, *J* = 24.9 Hz), 33.5–32.2 (m), 28.7–27.4 (m) ^19^F NMR (376 MHz, CDCl_3_) δ −185.1.

#### (2,3-Dimethoxyphenyl)(1-(4-fluorophenethyl)piperidin-4-yl)methanol [(±)MDL100907] (**11**)

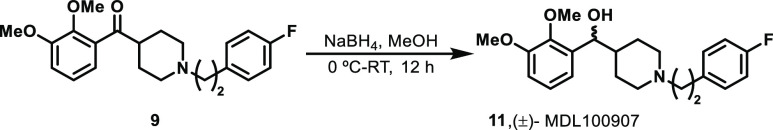



To a stirred solution of (2,3-dimethoxyphenyl)[1-[2-(4-fluorophenyl)ethyl]-4-piperidinyl] methanone **9** (100 mg, 0.26 mmol, 1 equiv) in MeOH (0.2 mL) at 0 °C was added NaBH_4_ (20 mg, 0.53 mmol, 2 equiv) in two portions, over a one hour period. The reaction mixture was slowly warmed to room temperature and stirred overnight. After completion of the reaction, the solvent was removed under vacuum. The residue was dissolved in water (5 mL) and extracted with Et_2_O (3 × 10 mL). The combined organic layer was dried using Na_2_SO_4_, filtered, and then concentrated under vacuum. The crude reaction mixture was purified by column chromatography to give (±) MDL100907 as a white solid (82 mg, 82% yield). *R*_f_ = 0.3 (EtOAc/hexane, 5:5); ^1^H NMR (300 MHz, CDCl_3_) δ 7.16–7.09 (m, 2H), 7.07–6.99 (m, 1H), 6.98–6.88 (m, 3H), 6.83 (dd, *J* = 8.1, 1.5 Hz, 1H), 4.66 (d, *J* = 7.5 Hz, 1H), 3.85 (s, 6H), 3.17 (d, *J* = 12.5 Hz, 1H), 3.04 (d, *J* = 11.2 Hz, 1H), 2.84 (dd, *J* = 10.6, 5.7 Hz, 2H), 2.63 (dd, *J* = 10.2, 6.2 Hz, 2H), 2.19–1.99 (m, 3H), 1.79–1.43 (m, 3H), 1.40–1.29 (m Hz, 1H); ^13^C NMR (75 MHz, CDCl_3_) δ 163.0, 159.8, 152.4, 146.3, 136.2, 135.1, 129.8 (d, *J* = 43.9 Hz), 123.7 (d, *J* = 41.7 Hz), 119.8 (d, *J* = 37.3 Hz), 116.0–114.0 (m), 112.4–110.2 (m), 75.0–72.5 (m), 61.2–59.3 (m), 55.8 (d, *J* = 23.5 Hz), 54.7–52.5 (m), 42.8–41.8 (m), 32.1, 28.4–27.6. ^19^F NMR (376 MHz, CDCl_3_) δ −104.19, −172.21.

#### (2,3-Dimethoxyphenyl)(1-(4-iodophenethyl)piperidin-4-yl)methanone (**10**)

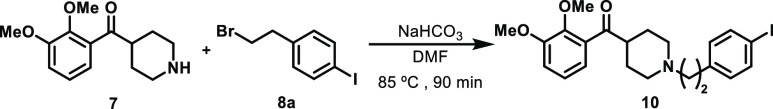



To a stirred suspension of (2,3-dimethoxyphenyl)(piperidin-4-yl)methanone **7** (1 g, 4.0 mmol, 1 equiv) and NaHCO_3_ (500 mg, 6 mmol, 1.5 equiv) in DMF (5 mL) was added 1-(2-bromoethyl)-4-iodobenzene (1.5 g, 4.8 mmol, 1.2 equiv) under a N_2_ atmosphere, and the reaction mixture was heated at 85 °C for 90 min. After the starting material disappeared, the reaction mixture was cooled to room temperature and water was added. Then, the mixture was extracted with ethyl acetate (3 × 50 mL). The combined organic layers were dried over anhydrous Na_2_SO_4_ and concentrated under reduced pressure. The residue was purified by silica gel chromatography to give **10** as a colorless oil (1.7 g, 91% yield). R_*f*_ = 0.3 (EtOAc/hexane, 7:3); ^1^H NMR (300 MHz, CDCl_3_) δ 7.61–7.54 (m, 2H), 7.12–6.91 (m, 5H), 3.89 (s, 3H), 3.85 (s, 3H), 3.08 (tt, *J* = 10.9, 3.9 Hz, 1H), 2.96 (dt, *J* = 11.5, 3.3 Hz, 2H), 2.77–2.69 (m, 2H), 2.59–2.49 (m, 2H), 2.11 (td, *J* = 11.4, 2.5 Hz, 2H), 1.95–1.85 (m, 2H), 1.81–1.65 (m, 2H). ^13^C NMR (75 MHz, CDCl_3_) δ 206.4, 152.7, 146.9, 140.1, 137.4 (d, *J* = 9.8 Hz), 134.3, 130.7 (d, *J* = 21.9 Hz), 124.5 (d, *J* = 24.0 Hz), 120.1 (d, *J* = 25.2 Hz), 114.9 (d, *J* = 25.2 Hz), 91.1, 61.9 (d, *J* = 20.0 Hz), 60.3, 56.0 (d, *J* = 20.3 Hz), 53.1 (d, *J* = 19.1 Hz), 48.1 (d, *J* = 14.3 Hz), 33.1, 28.0.

#### (2,3-Dimethoxyphenyl)(1-(4-iodophenethyl)piperidin-4-yl)methanol (**12**)

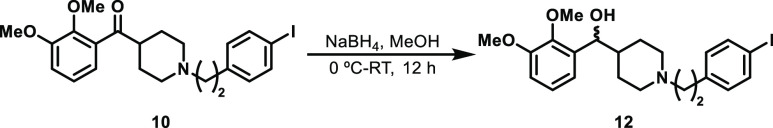



To a stirred solution of (2,3-dimethoxyphenyl) (1-(4-iodophenethyl)piperidin-4-yl)methanone **11** (1.5 g, 3.1 mmol, 1 equiv) in MeOH (2 mL) at 0 °C was added NaBH_4_ (0.230 g, 6.2 mmol, 2 equiv) in two portions, over a 1 h period. The reaction mixture was slowly warmed to room temperature and stirred overnight. After completion of the reaction, the solvent was removed under vacuum. The residue was dissolved in water (5 mL) and extracted with Et_2_O (3 × 100 mL). The combined organic layer was dried using Na_2_SO_4_, filtered, and then concentrated under vacuum. The crude reaction mixture was purified by column chromatography to give **12** as a colorless oil (1.2 g, 80% yield). *R*_f_ = 0.3 (EtOAc/hexane, 5:5); ^1^H NMR (300 MHz, CDCl_3_) δ 8.09–7.98 (m, 2H), 7.57–7.44 (m, 1H), 7.44–7.26 (m, 4H), 5.08 (d, *J* = 7.6 Hz, 1H), 4.36–4.30 (m, 6H), 3.51 (d, *J* = 10.0 Hz, 1H), 3.36 (d, *J* = 10.7 Hz, 1H), 3.28–3.13 (m, 2H), 3.06–2.90 (m, 2H), 2.53 (d, *J* = 13.1 Hz, 1H), 2.48–2.27 (m, 3H), 2.02–1.69 (m, 3H); ^13^C NMR (75 MHz, CDCl_3_) δ 152.9, 146.9, 140.6, 137.9 (d, *J* = 4.0 Hz), 137.7, 136.8, 132.0–130.5 (m), 125.0–123.7 (m), 120.6–119.3 (m), 111.6 (d, *J* = 24.0 Hz), 91.4, 74.8 (q, *J* = 47.3 Hz), 61.7–60.4 (m), 56.0 (d, *J* = 11.8 Hz), 54.1, 43.2, 33.7, 29.2.

#### (*R*)-(2,3-Dimethoxyphenyl)(1-(4-iodophenethyl)piperidin-4-yl)methyl 2-methoxy-2-phenylacetate (**14a**)

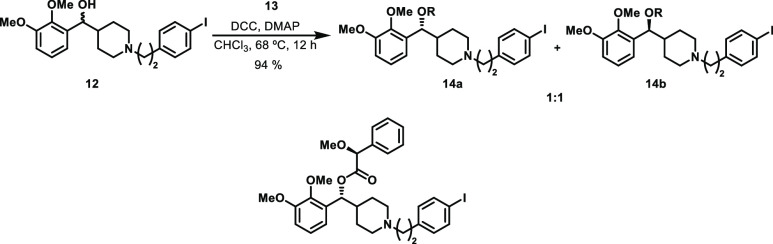



(2,3-Dimethoxyphenyl)(1-(4-iodophenethyl)piperidin-4-yl)methanol **12** (0.832 g, 1.7 mmol, 1 equiv) was dissolved in CHCl_3_ (10 mL) and added (*S*)-(+)-methoxyphenylacitic acid (0.287 g, 1.7 mmol, 1 equiv), dicyclohexylcarbodiimide (DCC) (0.350 g, 1.7 mmol, 1 equiv), and 4-*N*,*N*-dimethylaminopyridine (DMAP) (20 mg, 0.17 mmol, 0.1 equiv) sequentially under N_2_ at room temperature. The reaction mixture was stirred at 68 °C for 15 h, cooled to room temperature, and filtered. The solid was rinsed with ether. The filtrate was collected and concentrated under vacuum. The crude diastereomers were separated by column chromatography. The fraction containing the first-eluting diastereomeric ester was combined and evaporated to give **14a** as a white solid (500 mg, 42% yield). *R*_f_ = 0.5 (EtOAc/hexane, 5:5); ^1^H NMR (300 MHz, CDCl_3_) δ 7.58–7.52 (m, 2H), 7.48–7.42 (m, 2H), 7.40–7.31 (m, 3H), 6.97 (t, *J* = 8.0 Hz, 1H), 6.93–6.86 (m, 2H), 6.78 (ddd, *J* = 14.2, 8.0, 1.4 Hz, 2H), 5.90 (d, *J* = 8.3 Hz, 1H), 4.75 (s, 1H), 3.90 (s, 3H), 3.83 (s, 3H), 3.35 (s, 3H), 2.79 (d, *J* = 11.5 Hz, 2H), 2.64 (dd, *J* = 10.0, 6.0 Hz, 2H), 2.42 (dd, *J* = 9.7, 6.3 Hz, 2H), 1.84–1.72 (m, 2H), 1.71–1.58 (m, 1H), 1.44–1.26 (m, 2H), 1.25–1.01 (m, 2H); ^13^C NMR (75 MHz, CDCl_3_) δ 167.0, 152.4, 146.5, 140.1, 137.4, 137.2, 136.3, 132.9, 130.6 (d, *J* = 28.4 Hz), 128.7 (d, *J* = 8.5 Hz), 128.4 (d, *J* = 8.5 Hz), 127.1 (d, *J* = 21.5 Hz), 124.1 (d, *J* = 30.2 Hz), 118.7 (d, *J* = 22.2 Hz), 111.6 (d, *J* = 25.0 Hz), 91.0, 82.9 (q, *J* = 44.8 Hz), 75.0 (d, *J* = 23.2 Hz), 60.8–59.8 (m), 57.3 (t, *J* = 54.9 Hz), 55.5 (d, *J* = 22.6 Hz), 53.2 (d, *J* = 13.1 Hz), 41.0 (d, *J* = 15.1 Hz), 33.1, 27.4 (d, *J* = 42.4 Hz); HRMS (ESI) calcd for C_31_H_36_INO_5_ [M + H]^+^: 630.17184; found: 630.17117.

#### (*S*)-(2,3-Dimethoxyphenyl)(1-(4-iodophenethyl)piperidin-4-yl)methyl 2-methoxy-2-phenylacetate (**14b**)

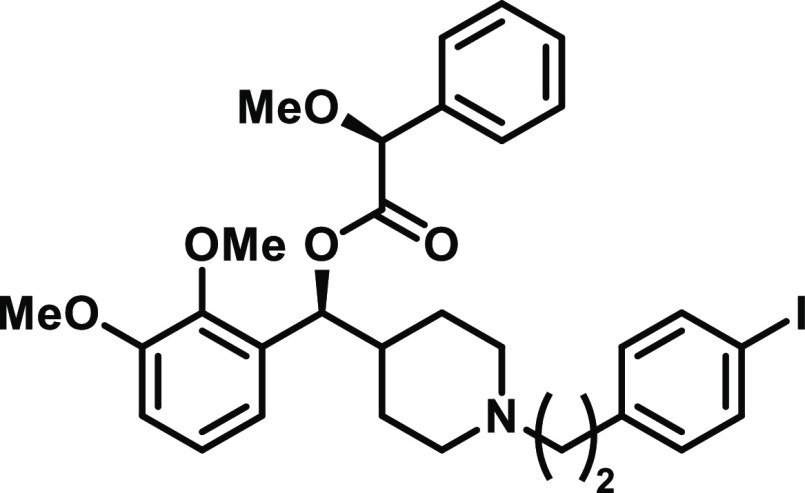



**14b** as a white solid (500 mg, 42% yield). *R*_f_ = 0.4 (AcOEt/hexane, 5:5); ^1^H NMR (300 MHz, CDCl_3_) δ 7.59–7.53 (m, 2H), 7.39–7.29 (m, 5H), 6.94–6.89 (m, 2H), 6.72–6.64 (m, 2H), 6.12 (dd, *J* = 6.5, 2.8 Hz, 1H), 5.94 (d, *J* = 6.8 H, 1H), 4.80 (s, 3H), 3.86 (s, 1H), 3.79 (s, 3H), 3.39 (s, 3H), 2.93 (t, *J* = 11.7 Hz, 2H), 2.70 (dd, *J* = 10.3, 5.7 Hz, 2H), 2.49 (dd, *J* = 9.9, 6.3 Hz, 2H), 1.96–1.80 (m, 2H), 1.70 (d, *J* = 8.7 Hz, 2H), 1.54–1.31 (m, 3H); ^13^C NMR (75 MHz, CDCl_3_) δ 169.7, 152.2, 146.1, 140.0, 137.4 (d, *J* = 11.6 Hz), 136.0, 132.6, 131.1, 130.5, 129.2–128.6 (m), 128.5–127.8 (m), 127.6–126.8 (m), 124.5–122.4 (m), 118.2 (d, *J* = 30 Hz), 112.0–110.1 (m), 91.08, 82.7 (d, *t* = 40 Hz), 74.5 (d, *J* = 25 Hz), 60.5–59.7 (m), 57.3 (d, *t* = 52 Hz), 55.7 (d, *J* = 24.3 Hz), 54.2–52.6 (m), 41.4–40.5 (m), 33.8–32.7 (m), 28.3–27.3 (m).

#### (*R*)-(2,3-Dimethoxyphenyl)(1-(4-(4,4,5,5-tetramethyl-1,3,2-dioxaborolan-2-yl)phenethyl) piperidin-4-yl)methyl(*S*)-2-methoxy-2-phenylacetate (**15**)

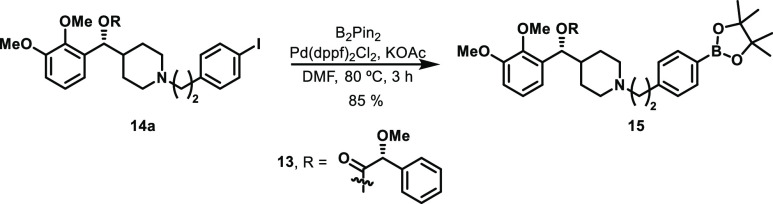



(1,1′-Bis(diphenylphosphino)ferrocene)-palladium(II) dichloride (6 mg, 0.02 mmol, 0.03 equiv) was added to a degassed (15 min nitrogen bubbling) mixture of **14a** (0.600 g, 0.9 mmol, 1 equiv), bispinacolatodiboron (50 mg, 0.2 mmol, 1.1 equiv), and potassium acetate (52 mg, 0.5 mmol, 1.3 equiv) in DMF (10 mL). The reaction mixture was stirred for 3 h at 80 °C under a nitrogen atmosphere. The reaction mixture was cooled to room temperature, quenched with brine, and extracted with ethyl acetate (3 × 100). The organic layers were washed with brine, dried over sodium sulfate, filtered, and concentrated. The resulting crude reaction mixture was purified by column chromatography (SiO_2_ was neutralized with Et_3_N before loading the crude mixture). After concentration under reduced pressure, the precursor **15** was isolated as a tan solid (0.510 g, 85% yield). *R*_f_ = 0.3 (DCM: MeOH, 8: 2); ^1^H NMR (300 MHz, CDCl_3_) δ 7.70 (d, *J* = 8.0 Hz, 2H), 7.49–7.42 (m, 2H), 7.40–7.31 (m, 3H), 7.19–7.13 (m, 2H), 7.01–6.93 (m, 1H), 6.78 (ddd, *J* = 15.1, 8.0, 1.4 Hz, 2H), 5.89 (d, *J* = 8.2 Hz, 1H), 4.75 (s, 1H), 3.90 (s, 3H), 3.84 (s, 3H), 3.36 (s, 3H), 2.82 (d, *J* = 11.0 Hz, 2H), 2.73 (dd, *J* = 9.9, 6.3 Hz, 2H), 2.47 (dd, *J* = 9.9, 6.3 Hz, 2H), 1.85–1.73 (m, 2H), 1.72–1.62 (m, 1H), 1.44–1.35 (m, 2H), 1.32 (s, 12H), 1.21–1.12 (m, 2H). ^13^C NMR (75 MHz, CDCl_3_) δ 170.0, 152.4, 146.6, 143.9, 136.3, 134.9, 133.0, 129.2–128.2 (m), 127.9, 127.4, 127.1, 124.3–123.6 (m), 118.7 (d, *J* = 20 Hz), 111.6 (d, *J* = 22.8 Hz), 83.6, 83.5, 82.9, 82.6, 75.2–74.6 (m), 60.3 (d, *J* = 22.0 Hz), 57.3 (t, *J* = 22.8 Hz), 55.5 (d, *J* = 22.0 Hz), 53.8–52.6 (m), 41.2 (d, *J* = 15.0 Hz), 33.8, 28.3–27.1 (m), 25.11, 24.93, 24.73, 24.54.; HRMS (ESI) calcd for C_37_H_48_BNO_7_ [M + H]^+^: 629.36329; found: 629.36401.

### Radiochemistry

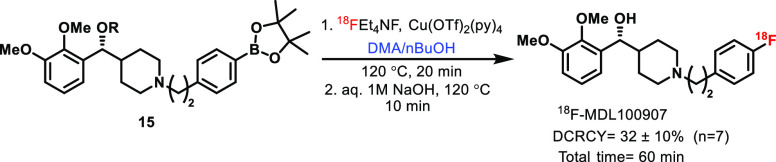



To a glass vessel containing a solution of MeCN (1.0 mL), was eluted ^18^F-fluoride 18.5 Bq (∼0.500 Ci) from an anion-exchange resin (QMA cartridge, 46 mg) with a solution of Et_4_NHCO_3_ (1.5 mg) in H_2_O (600 μL). The solvent was removed at 120 °C with a nitrogen flow, and additional MeCN (3.5 mL) was added, followed by evaporation of the solvent with a nitrogen flow to remove residual H_2_O. A solution of precursor **15** (10 mg, 10 μmol) and Cu(OTf)_2_(py)_4_ (21 mg, 10 μmol) in DMA/nBuOH (300/100 μL) was added, and the reaction mixture was heated at 120 °C for 20 min with stirring under air in a capped vial. Thereafter, the reactor was cooled to ∼40 °C and 0.5 mL of 1 M sodium hydroxide was added, and the mixture was allowed to react for 15 min at 120 °C. The mixture was then diluted with 6 mL of eluent (50%, H_2_O/EtOH, 0.1% TEA) and purified by prep HPLC (waters X Terra Prep RP18, 5 μm, 19 mm × 100 mm column) using water/EtOH/Et_3_N 50:50:0.01 (v/v/v) flow rate: 6 mL/min; *t*_R_ = 15.5 min. The collected fraction was diluted with enough water to reduce the total concentration of organic solvent below 10%. The solution was then loaded onto a preconditioned Sep-Pak C18 cartridge, which was washed with water (10 mL) and briefly dried by a steam of argon before the product was eluted with 1.5 mL of EtOH into dose vials containing 13.5 mL of saline and was ready for microPET studies. Evidence of the identity of [^18^F]MDL100907 was achieved by comparing the *R*_f_ of the radioactive product with the *R*_f_ of the authentic cold compound [^19^F]MDL100907 on analytical HPLC (waters X Terra Prep RP18, 5 μm, 7.8 mm × 100 mm column) using the solvent system water/EtOH/Et_3_N 50:50:0.01 (v/v/v); flow rate: 2 mL/min; *t*_R_ = 8 min (see Figures S2–S15). The pH of the final dose solution was tested with pH paper and found to be 6–7. The isolated radiochemical yield was 4 Bq (150 mCi) in 15 mL of 10% EtOH/saline as determined using a dose calibrator, affording a 32% decay-corrected radiochemical yield based on a synthesis time of approx. 60 min, which proceeded immediately upon the end of the cyclotron bombardment (Scheme 1).

### Nonhuman Primate Imaging

The microPET study was performed using an adult male rhesus monkey. All protocols, animal care, and handling followed the National Institutes of Health Guide for the Care and Use of Laboratory Animals (8th edition, revised 2011) and the recommendations of AAALAC International and were approved by Emory University’s Institutional Animal Care and Use Committee (IACUC). The animal was fasted for 12 h prior to the PET study. The animals were initially anesthetized with an intramuscular injection of Telazol (3 mg/kg), intubated, and then maintained on a 1% isoflurane/5% oxygen gas mixture throughout the imaging session. [^18^F]MDL100907 was injected *via* the antecubital vein over the course of 5 min. Quantitative brain image studies were performed using a Siemens MicroPET Focus 220 scanner. A transmission scan was obtained with a germanium-68 source prior to the PET study for attenuation correction of the emission data. The scan was conducted following the injection of [^18^F]MDL100907. Emission data were collected continuously in list mode for 120 min after injection of [^18^F]MDL100907 and then rebinned into a 24-frame dynamic sequence for analysis.

The same animal underwent two PET neuroimaging studies acquired on a microPET focus 220 scanner system (CTI Concorde Microsystems LLC, Knoxville, TN). Animal anesthesia (isoflurane 1–2% to effect) and monitoring followed standard veterinary practices approved by Emory’s Institutional Animal Care and Use Committee (IACUC). In Nov. 2015, 2.9 mCi of [^11^C]MDL100907 was administered intravenously over one minute beginning simultaneously with a 90 min PET emission scan. Later, in Dec. 2021, 5.5 mCi of [^18^F]MDL100907 was administered intravenously over one minute beginning simultaneously with a 120 min PET emission scan. PET emission data were binned into individual frames and reconstructed. Time–activity curves were generated for regions in the frontal cortex and mid-brain areas using in-house templates developed for rhesus macaques.

## Data Availability

The data will be available upon request.

## Supporting Information Available

Copies of ^1^H NMR, ^13^C NMR, and ^19^F NMR and HRMS spectra; [^18^F]MDL100907 (**11**) production data; radiometric and UV HPLC analysis of [^18^F]MDL100907 (**11**); and time–activity curves of regional brain uptake of [^18^F]MDL100907 (**11**) and [^11^C]MDL100907 (PDF)
