# Supplementary material for: Concise and Scalable Radiosynthesis of (+)-[18F]MDL100907 as a Serotonin 5-HT2A Receptor Antagonist for PET
Source: ACS Chem Neurosci. 2023 Sep 25;14(19):3694–703. doi: 10.1021/acschemneuro.3c00382 (PMC10557077; doi:10.1021/acschemneuro.3c00382)
Supplement: Supplementary file 1 — cn3c00382_si_001.pdf [file cn3c00382_si_001.pdf]

A Concise and Scalable Radiosynthesis of (+)-[^{18}F]MDL100907 as Serotonin 5-HT$_{2A}$ Receptor Antagonist for PET

Lahu N. Chavan,[1*] Ronald Voll,[1,2*] Mar M. Sanchez,[2,3] Jonathon A. Nye[1,2] and Mark M. Goodman[1,2,3*]
[*] These authors contributed equally to the work.
[1]Department of Radiology and Imaging Sciences, Center for Systems Imaging,
[2]Department of Psychiatry and Behavioral Sciences,
[3]Emory National Primate Center Emory University, Atlanta, Georgia 30322.

Supporting Information

Table of Contents

NMR spectra.. S-2−S-13

- Figure S1: ^{1}H and ^{13}C NMR, compound **4**………………………………………… S-2
- Figure S2: ^{1}H and ^{13}C NMR, compound **6**………………………………………… S-3
- Figure S3: ^{1}H and ^{13}C NMR, compound **7**………………………………………… S-4
- Figure S4: ^{1}H and ^{13}C NMR, compound **9**………………………………………… S-5
- Figure S5: ^{19}F NMR, compound **9**………………………………………… S-6
- Figure S6: ^{1}H and ^{13}C NMR, compound **11**………………………………………… S-7
- Figure S7: ^{19}F NMR, compound **11**………………………………………… S-8
- Figure S8: ^{1}H and ^{13}C NMR, compound **10**………………………………………… S-9
- Figure S9: ^{1}H and ^{13}C NMR, compound **12**………………………………………… S-10
- Figure S10: ^{1}H and ^{13}C NMR, compound **14a**………………………………………… S-11
- Figure S11: ^{1}H and ^{13}C NMR, compound **14b**………………………………………… S-12
- Figure S12: ^{1}H and ^{13}C NMR, compound **15**………………………………………… S-13

HRMS spectra ………………………………………… S-14−S 15

- Figure S13: HRMS compound **14a**………………………………………… S-14
- Figure S14: HRMS compound **15**………………………………………… . S-15

Radiometric chromatographs………………………………………… S-16−S-19

- Figure S15: Radiometric HPLC chromatogram of **[^{18}F]11** prep column purification………… S-16
- Figure S16 Radiometric HPLC chromatogram of **[^{18}F]11** crude reaction mixture………… S-16
- Figure S17 Radiometric and UV HPLC chromatogram of **[^{18}F]11** crude reaction mixture………… S-17
- Figure S18 Radiometric HPLC chromatogram of **[^{18}F]11** purified dose………… S-17
- Figure S19 Radiometric and UV HPLC HPLC chromatogram of **[^{18}F]11** purified dose………… S-18
- Figure S20 Radiometric chiral HPLC chromatogram of **[^{18}F]11** purified dose ………… S-18
- Figure S21 Radiometric and UV 280 nM chiral HPLC chromatogram of **[^{18}F]11** and racemic **11**………… S-19
- Figure S22 Radiometric and UV 254 nM and 280 nM chiral HPLC chromatogram of **[^{18}F]11** and racemic **1** S-19

Table 1. **[^{18}F]11** Production Runs ………… S-20

Figure S23 Calibration curve **[^{19}F]11** ………… S-21

MicroPET Image Data ………………………………………… S-22

Figure S24 Time activity curves of **[^{18}F]11** in a male rhesus monkey S-22

Figure S25 Time activity curves of **[^{11}C]11** in a male rhesus monkey S-22

…………………………………………

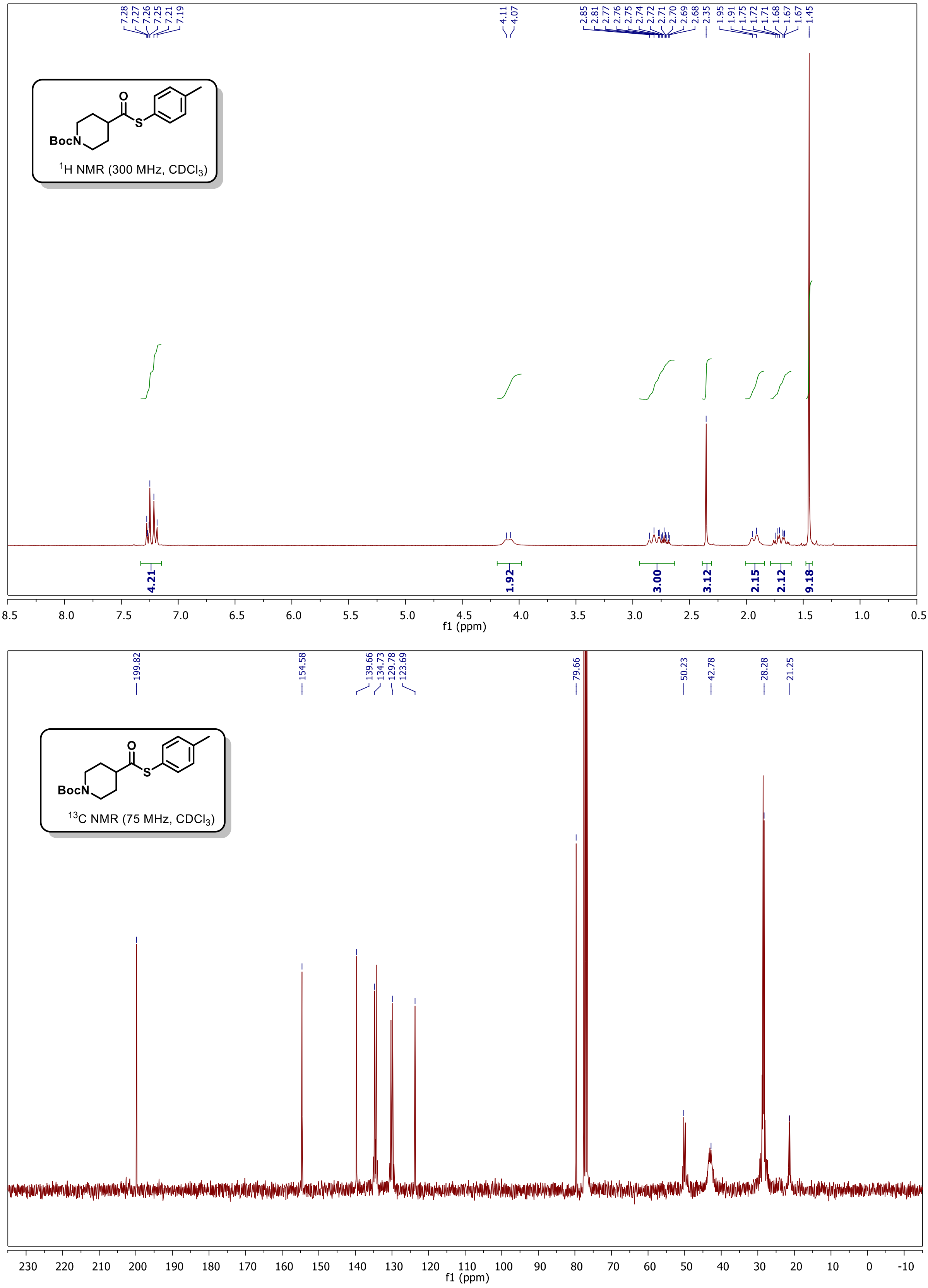

Figure S1. ^{1}HNMR (top) and ^{13}CNMR (bottom) of *tert*-butyl 4-((*p*-tolylthio)carbonyl)piperidine-1-carboxylate (**4**).

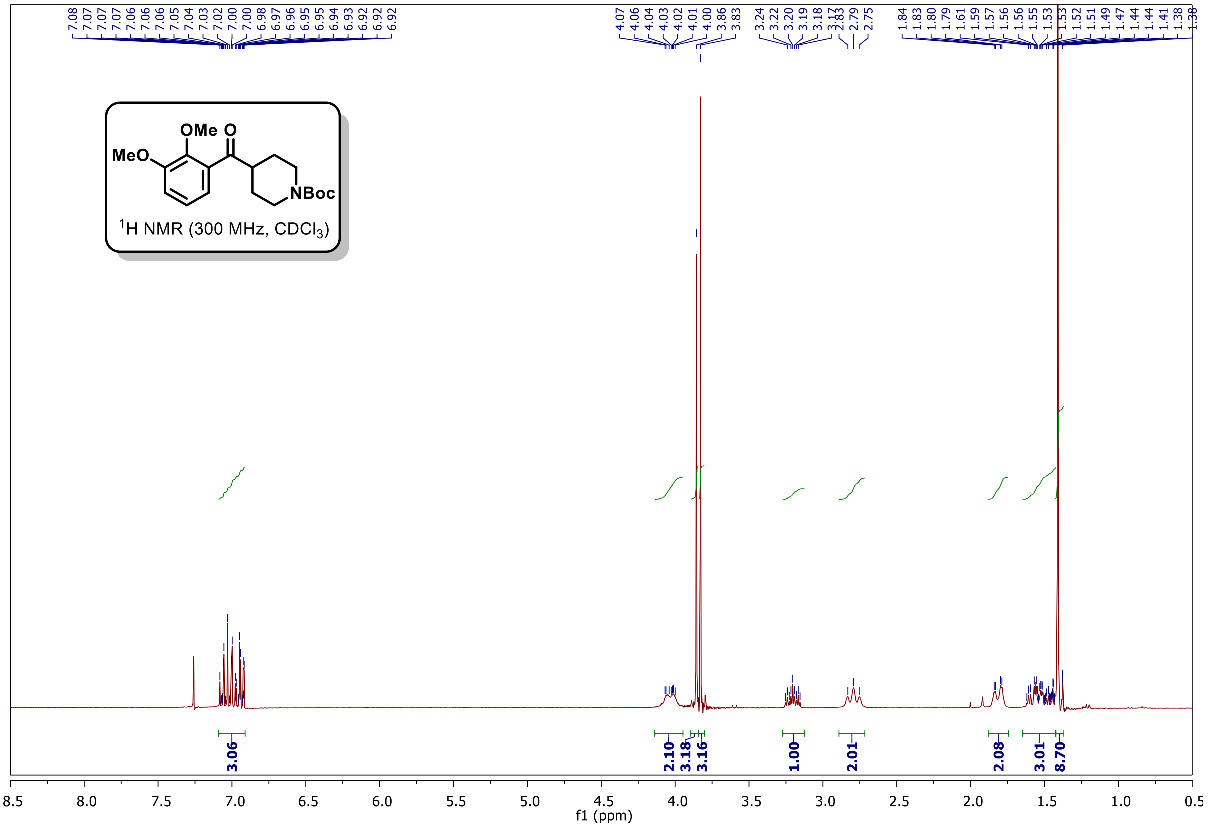

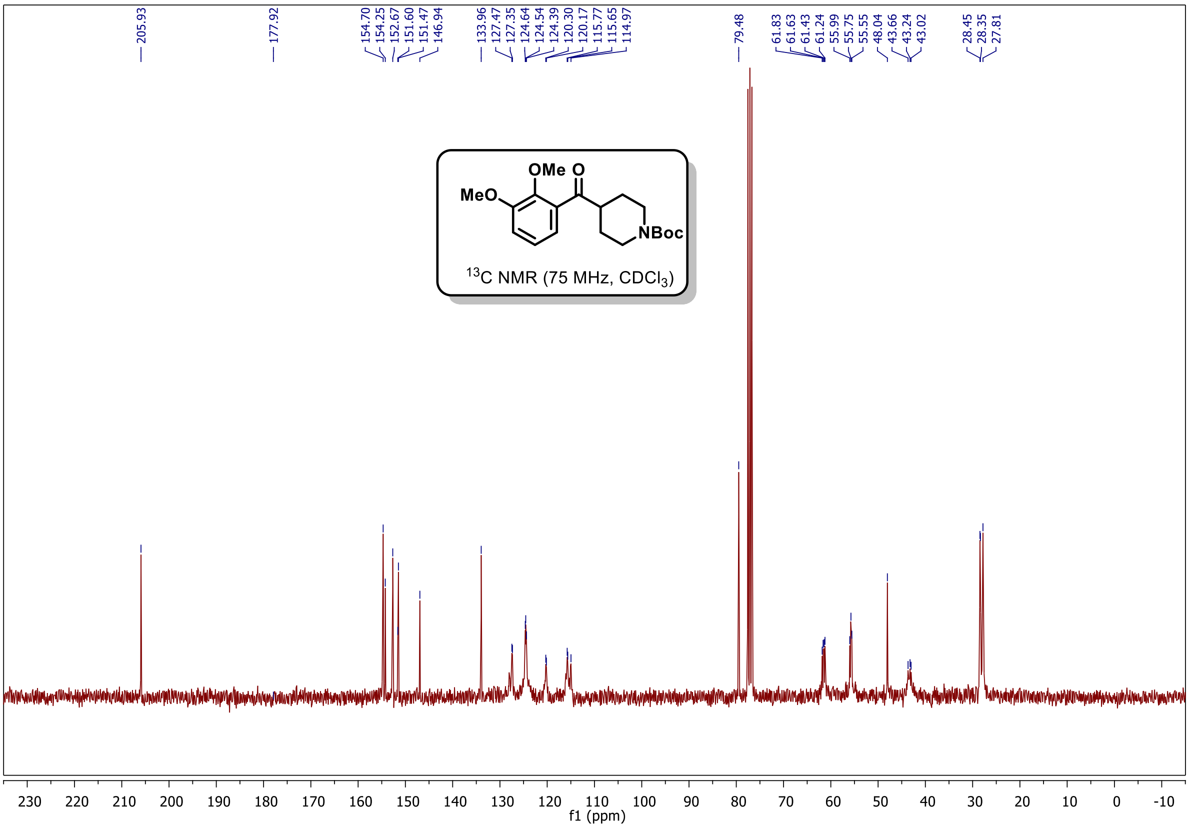

Figure S2. ^{1}HNMR (top) and ^{13}CNMR (bottom) of *tert*-butyl 4-(2, 3-dimethoxybenzoyl)piperidine-1-carboxylate **(6).**

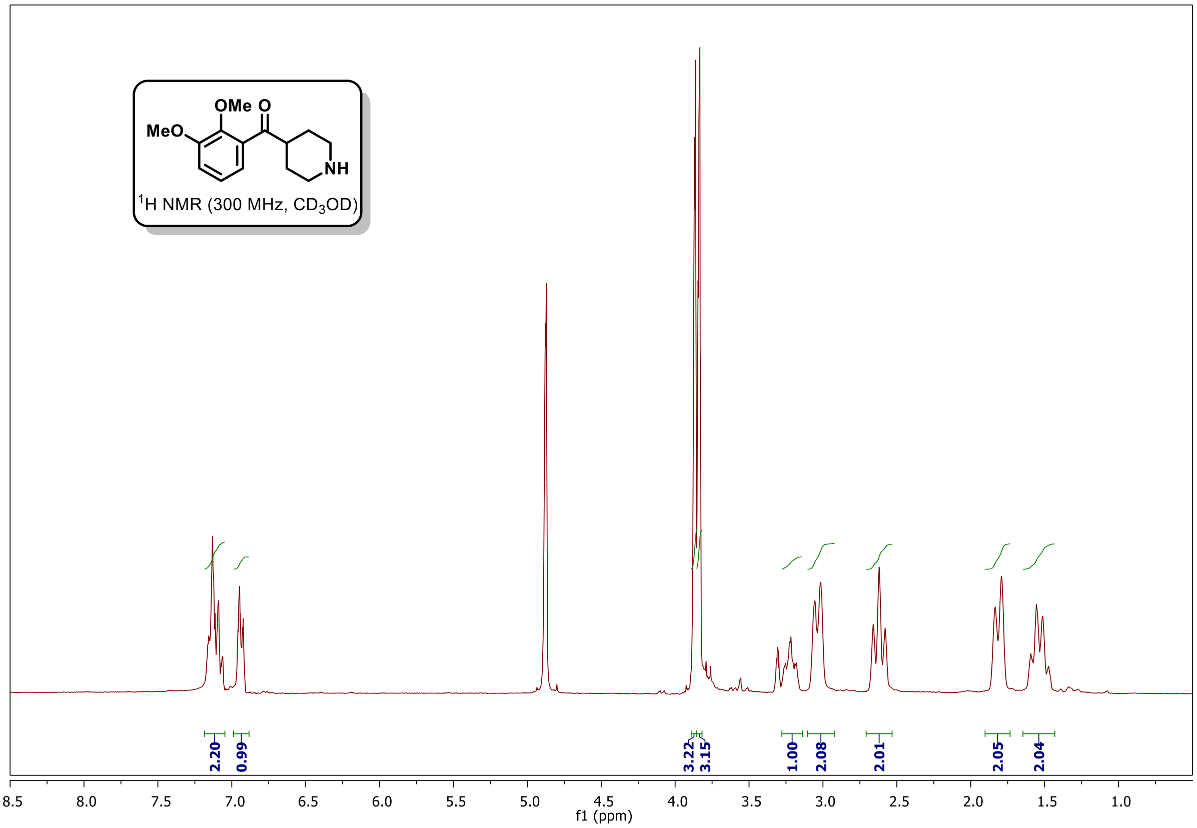

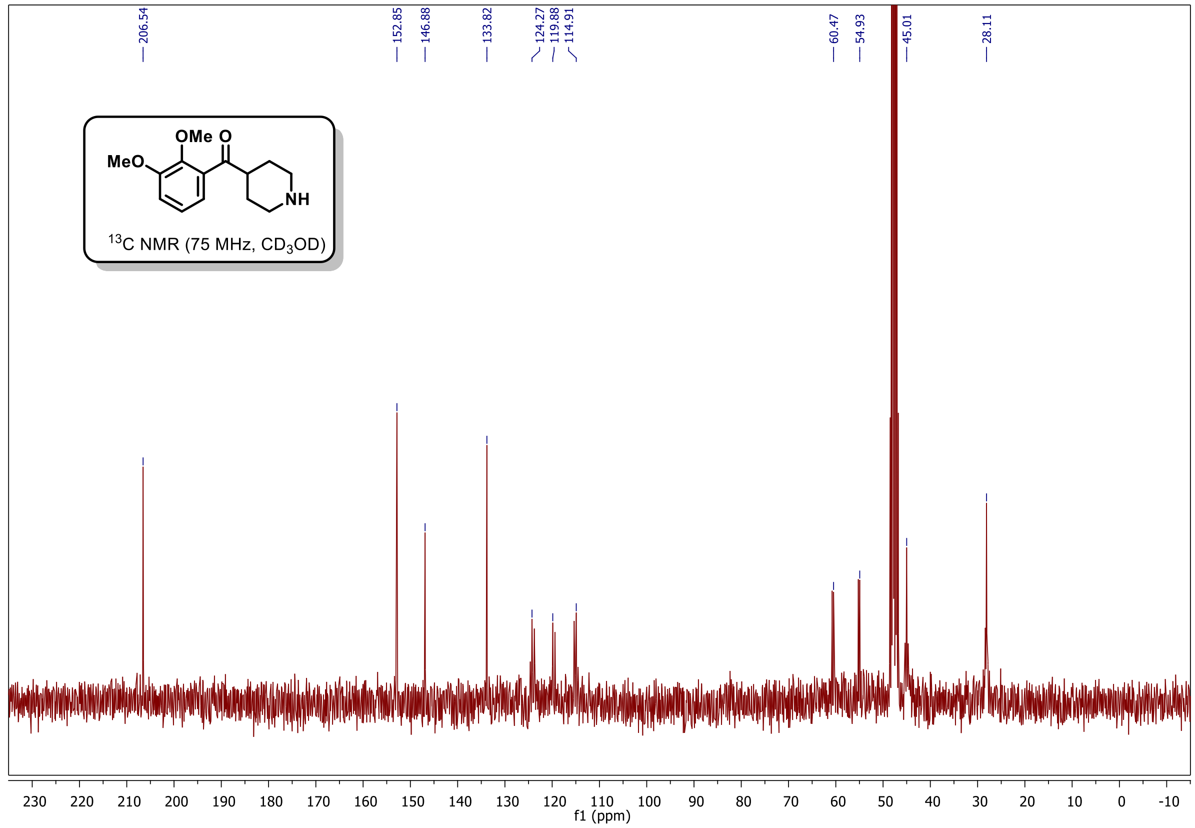

Figure S3. ^{1}HNMR (top) and ^{13}CNMR (bottom) of (2, 3 dimethoxyphenyl)(piperidin-4-yl)methanone **(7).**

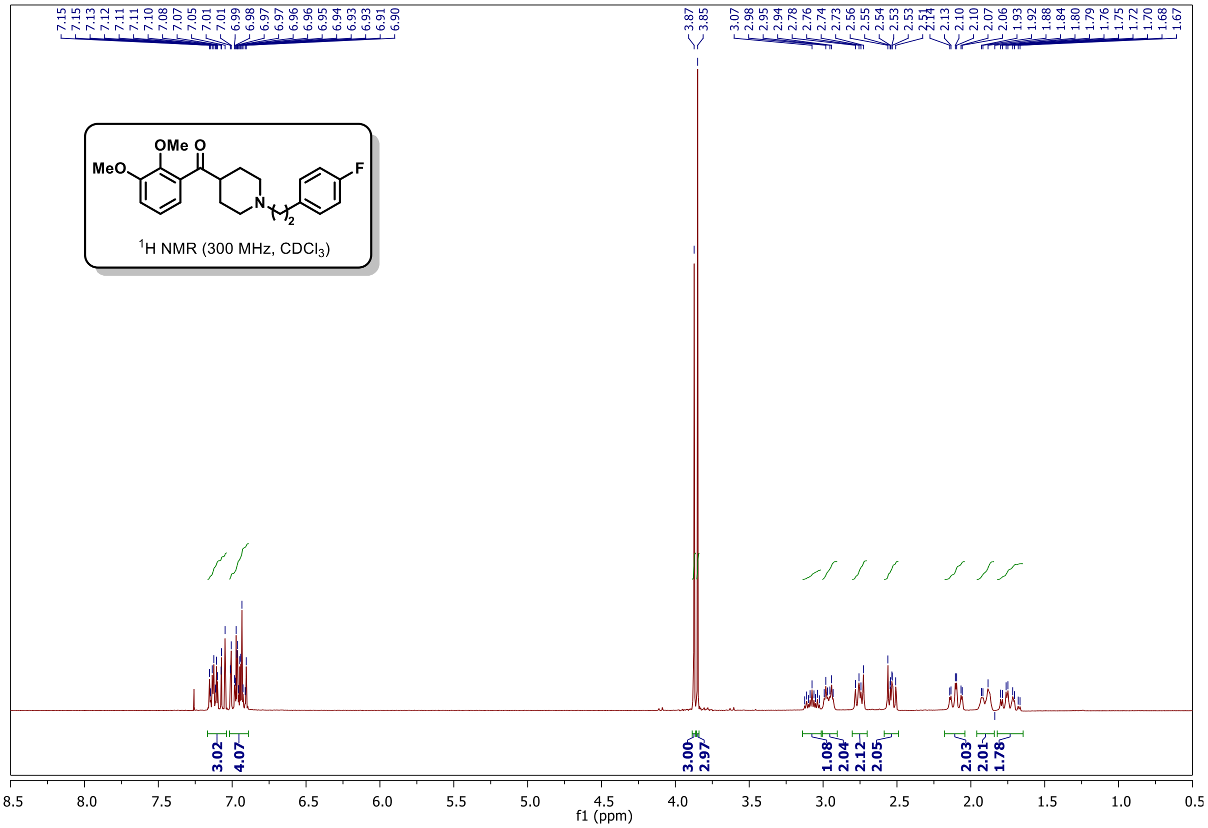

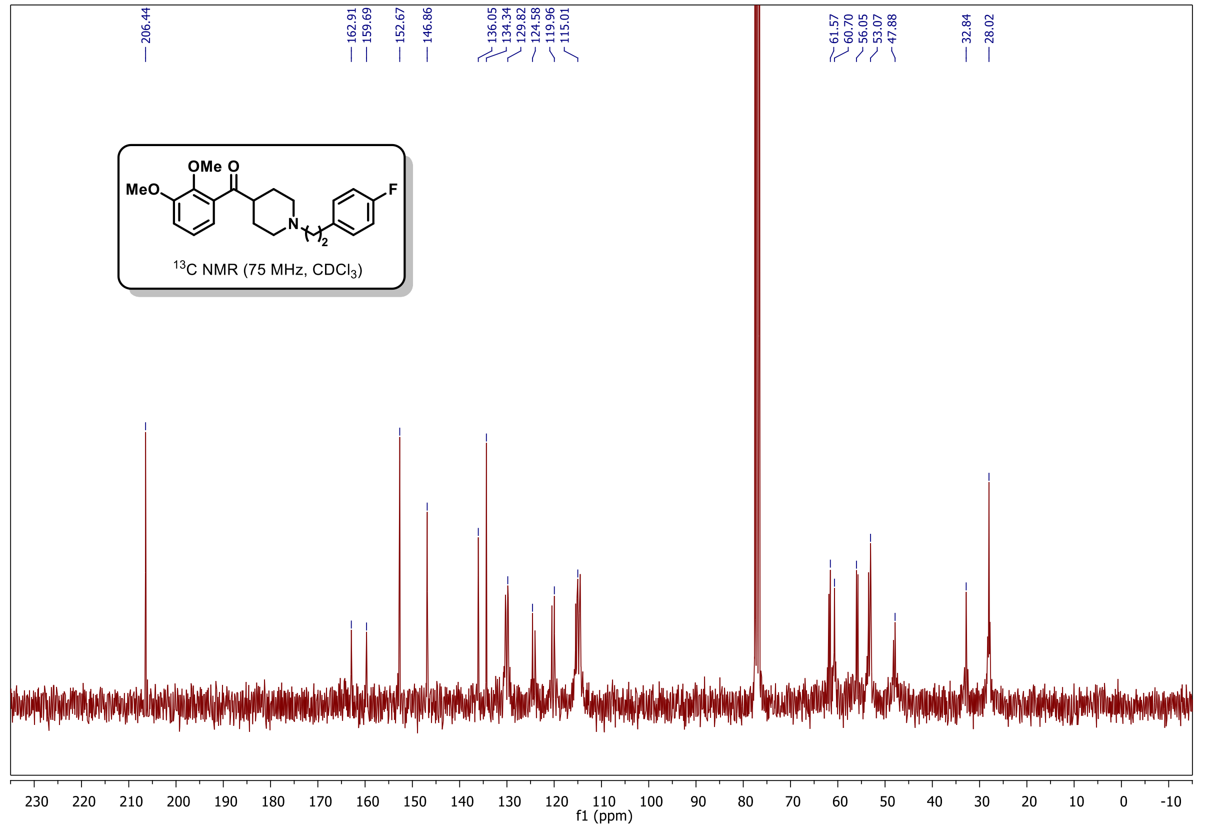

Figure S4. ^{1}HNMR (top) and ^{13}CNMR (bottom) of ((2, 3-dimethoxyphenyl)(1-(4 fluorophenethyl)piperidin-4-yl)methanone **(9).**

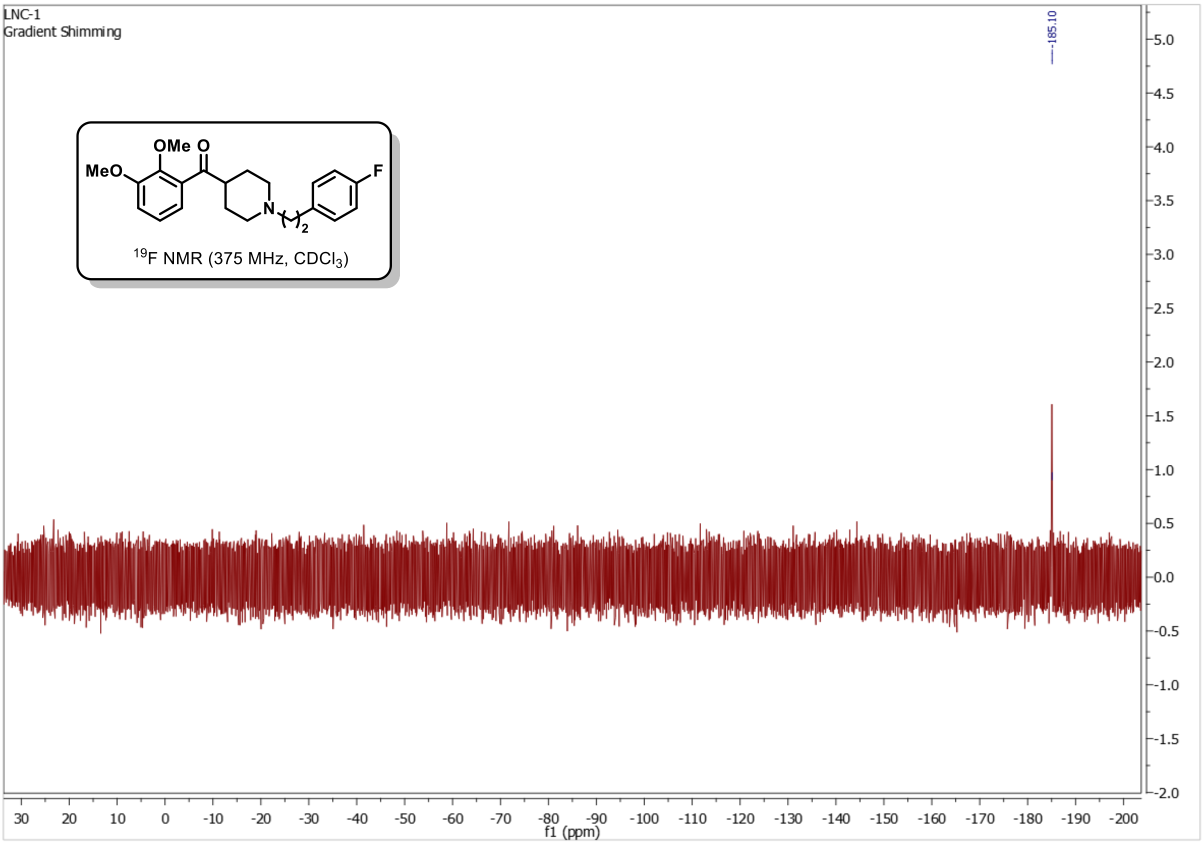

Figure S5. ^{19}FNMR of ((2, 3-dimethoxyphenyl)(1-(4-fluorophenethyl)piperidin-4-yl)methanone **(9).**

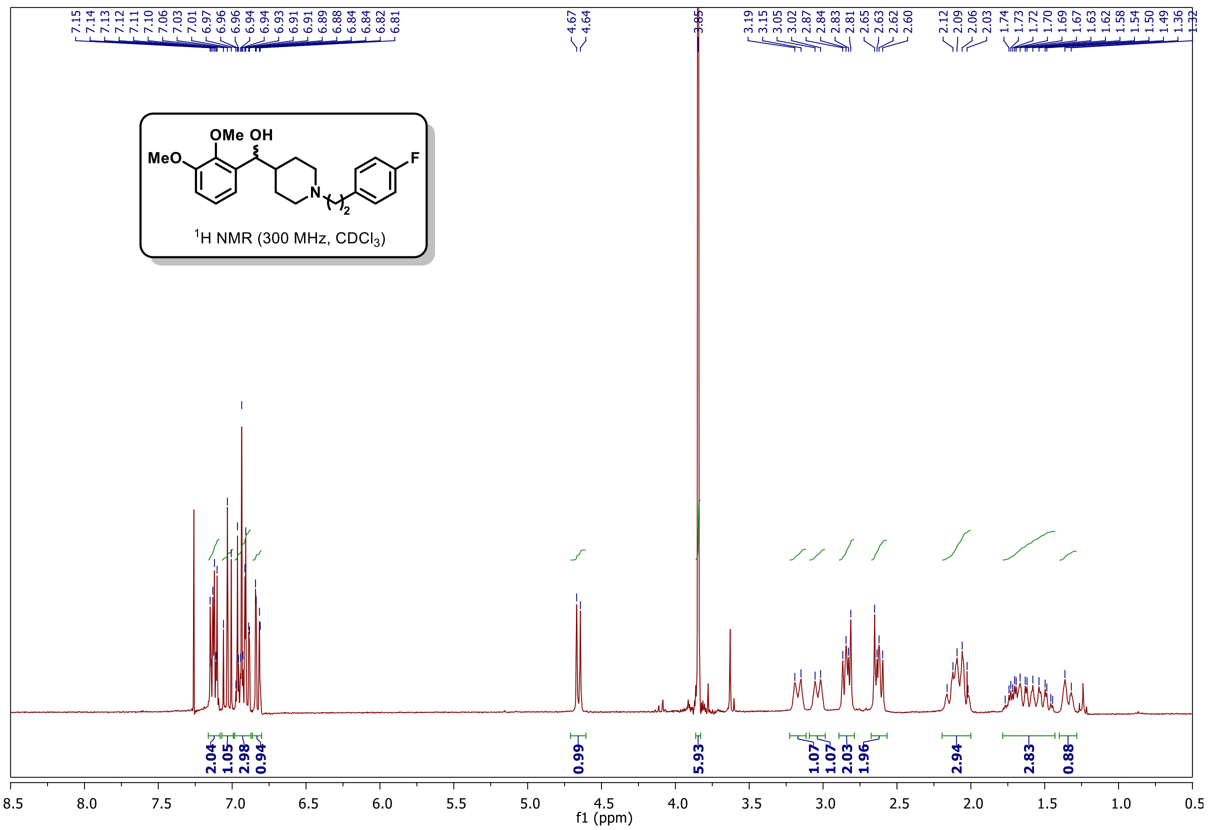

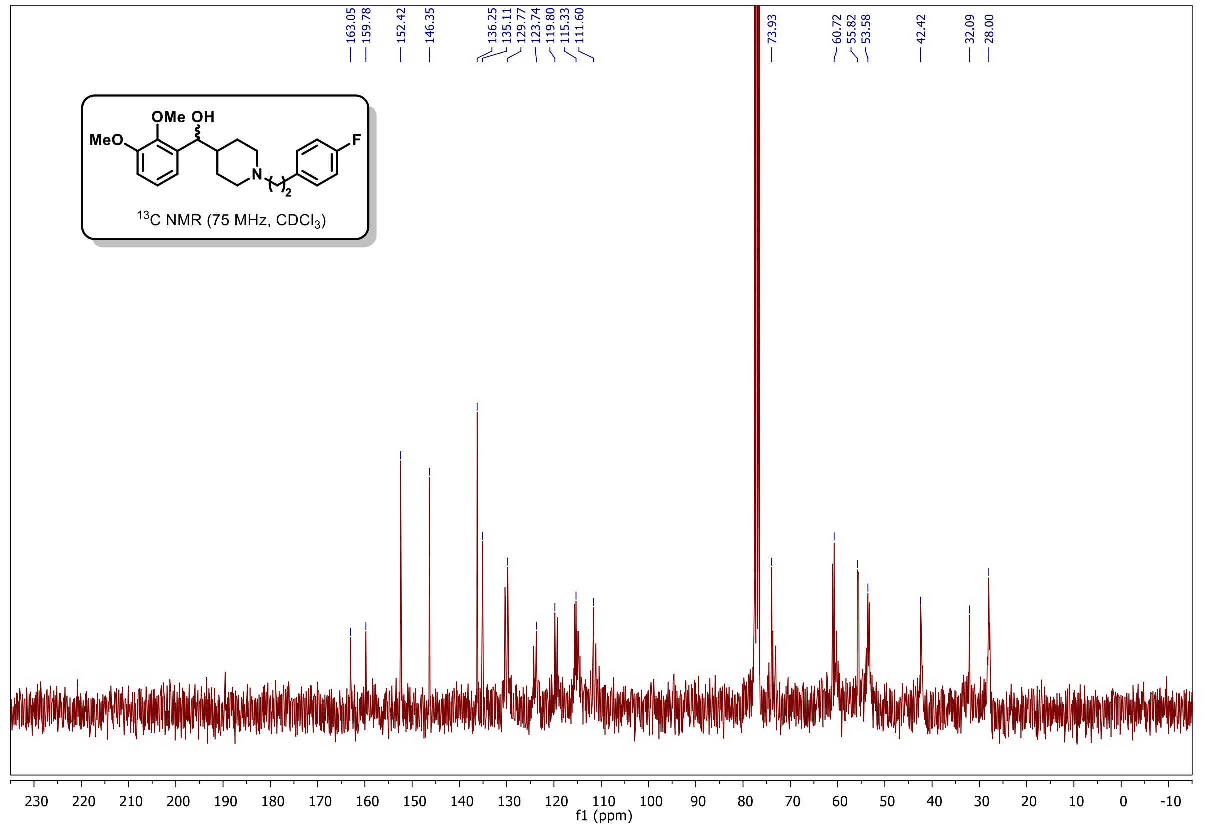

Figure S6. ^{1}HNMR (top) and ^{13}CNMR (bottom) of (2, 3-dimethoxyphenyl)(1-(4-fluorophenethyl)piperidin-4-yl)methanol [(±)MDL100907] **(11).**

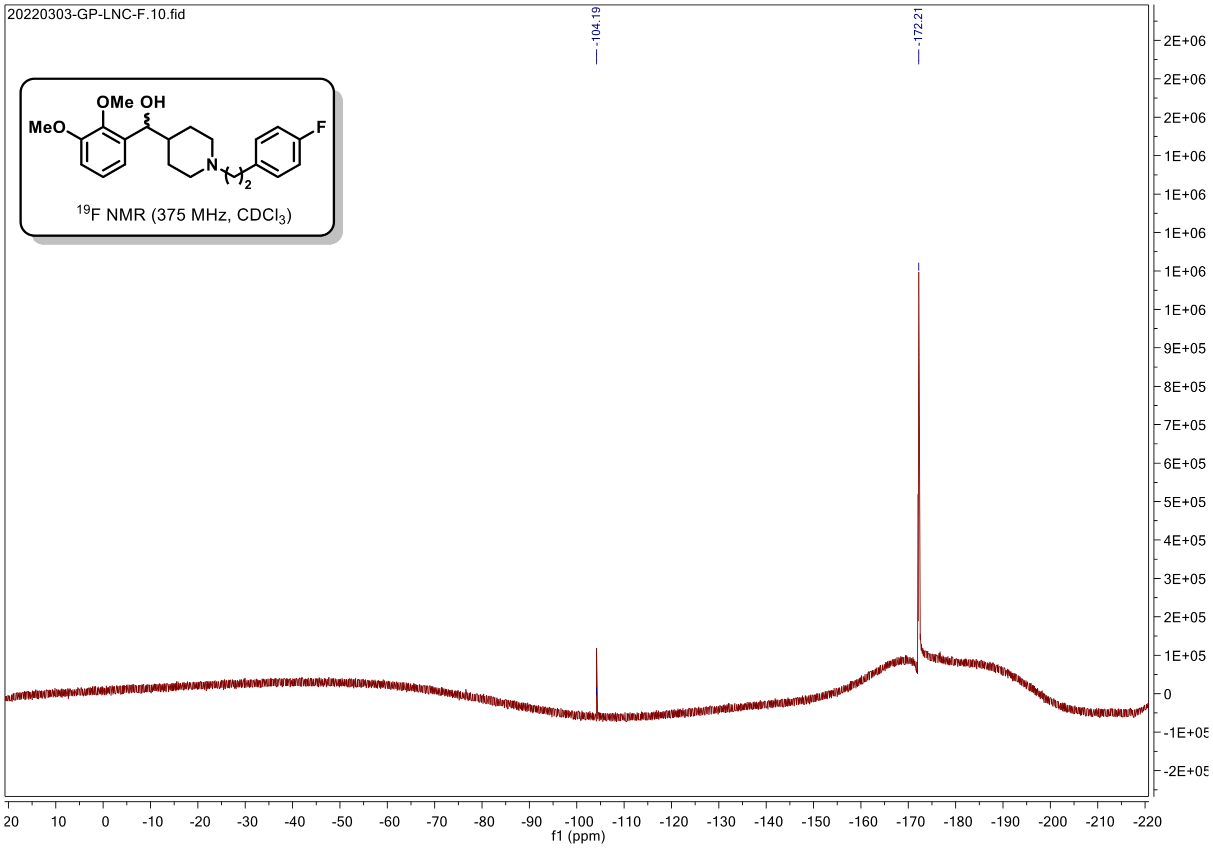

Figure S7. ^{19}FNMR of (2, 3-dimethoxyphenyl)(1-(4-fluorophenethyl)piperidin-4-yl)methanol [(±)MDL100907] **(11).**

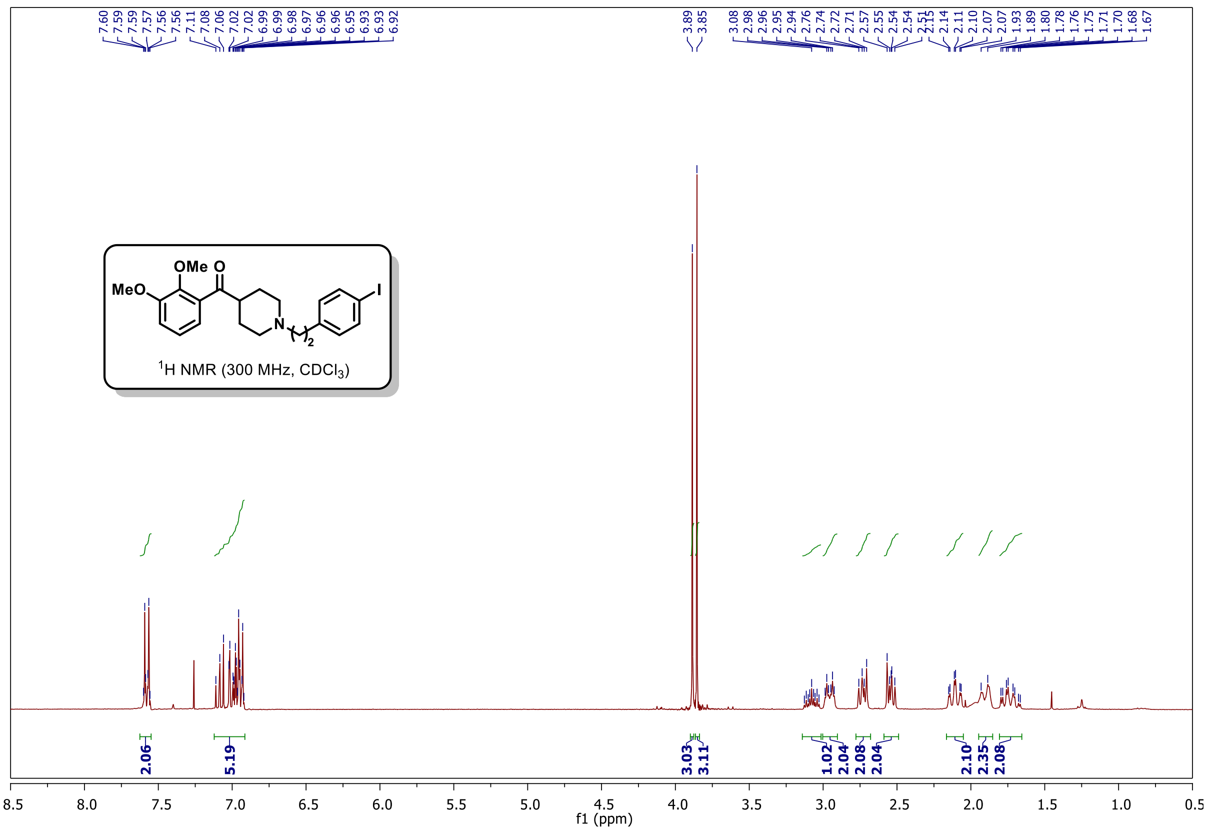

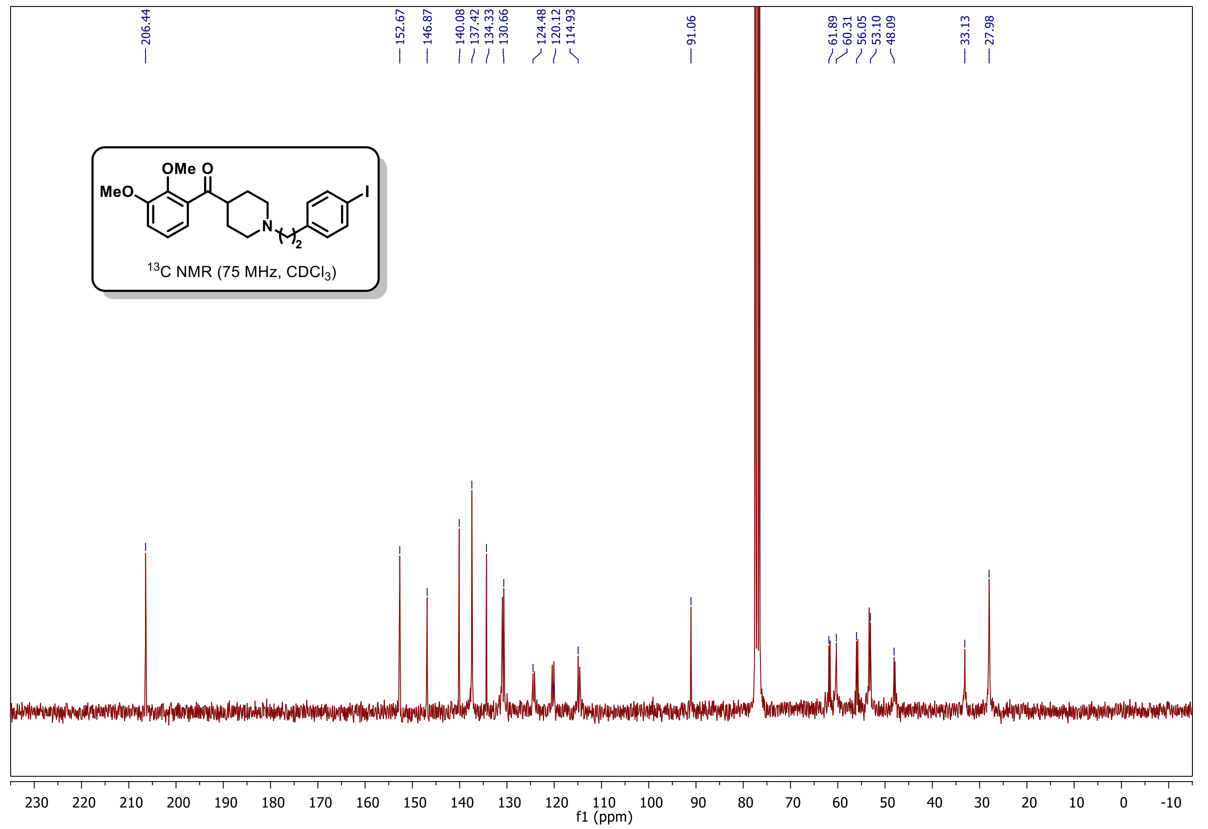

Figure S8. ^{1}HNMR (top) and ^{13}CNMR (bottom) of (2, 3-dimethoxyphenyl)(1-(4-iodophenethyl)piperidin-4-yl)methanone **(10).**

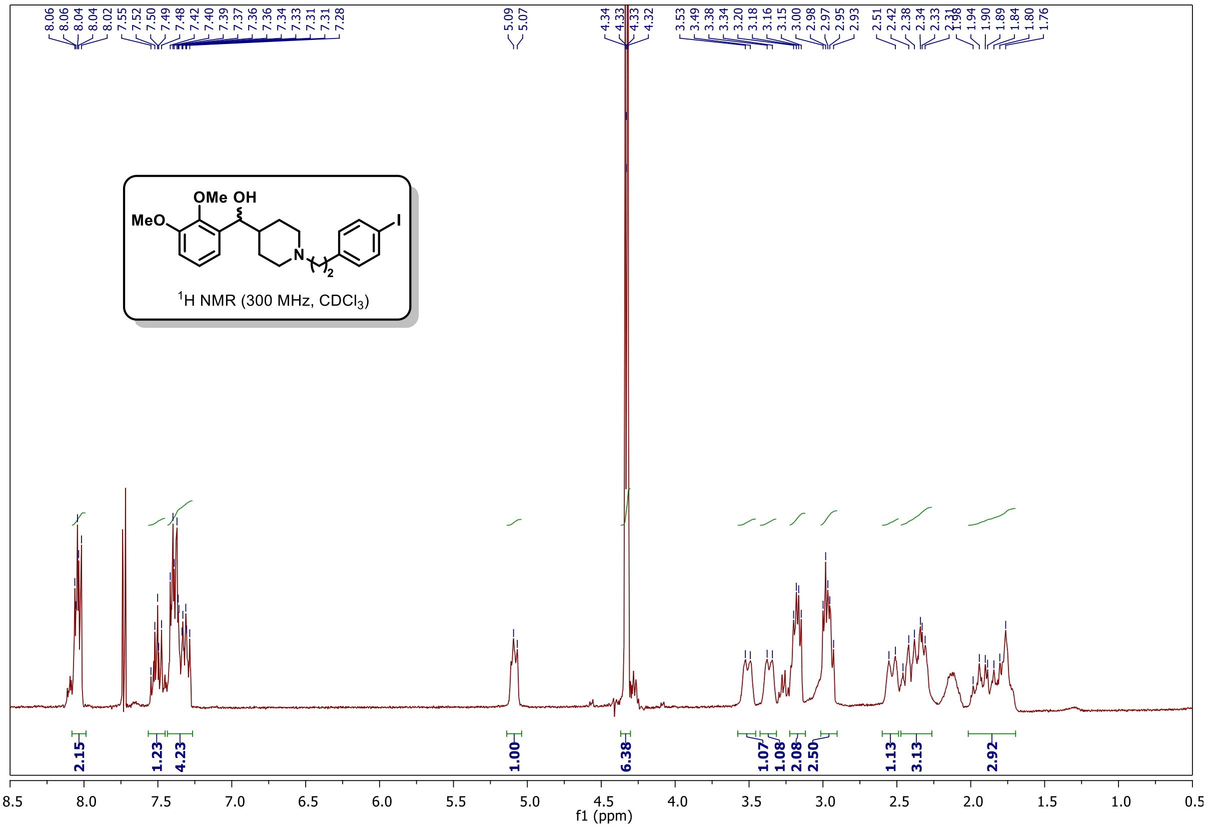

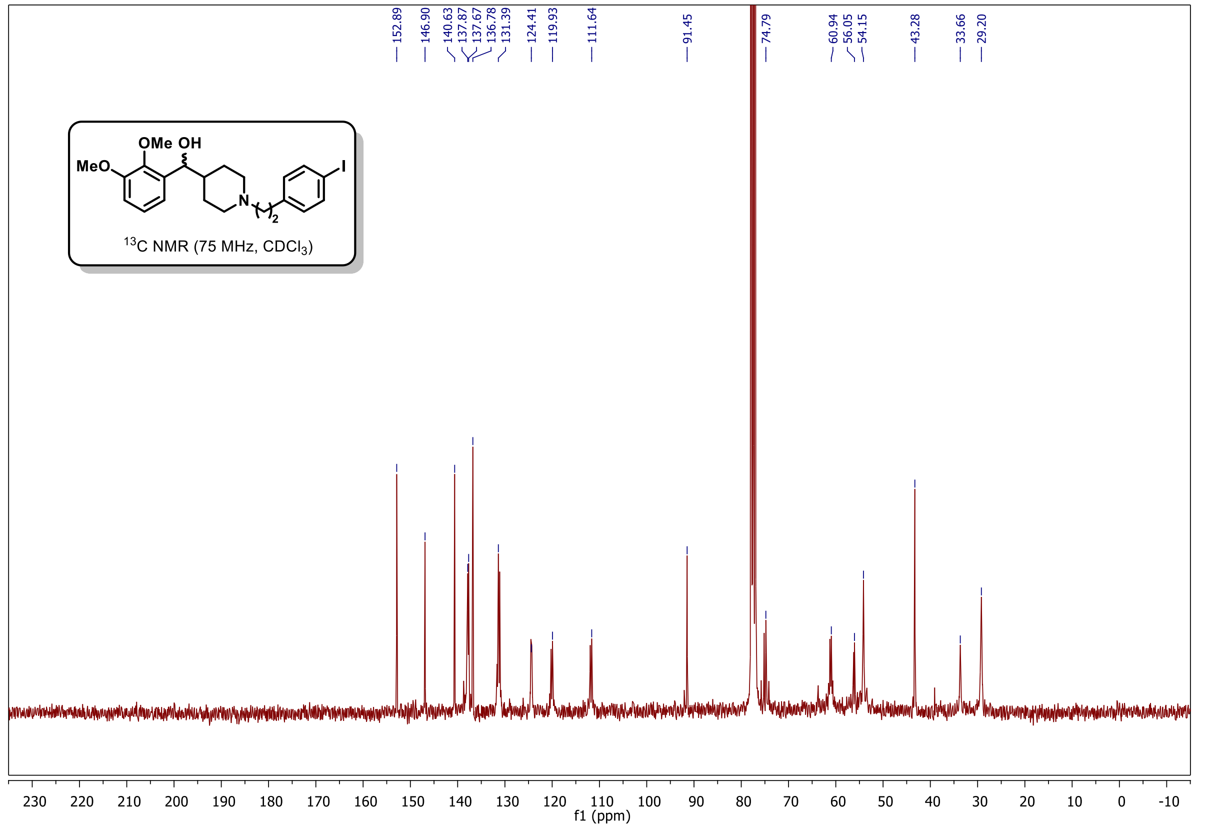

Figure S9. ^{1}HNMR (top) and ^{13}CNMR (bottom) of (2, 3-dimethoxyphenyl)(1-(4-iodophenethyl)piperidin-4-yl)methanol **(12).**

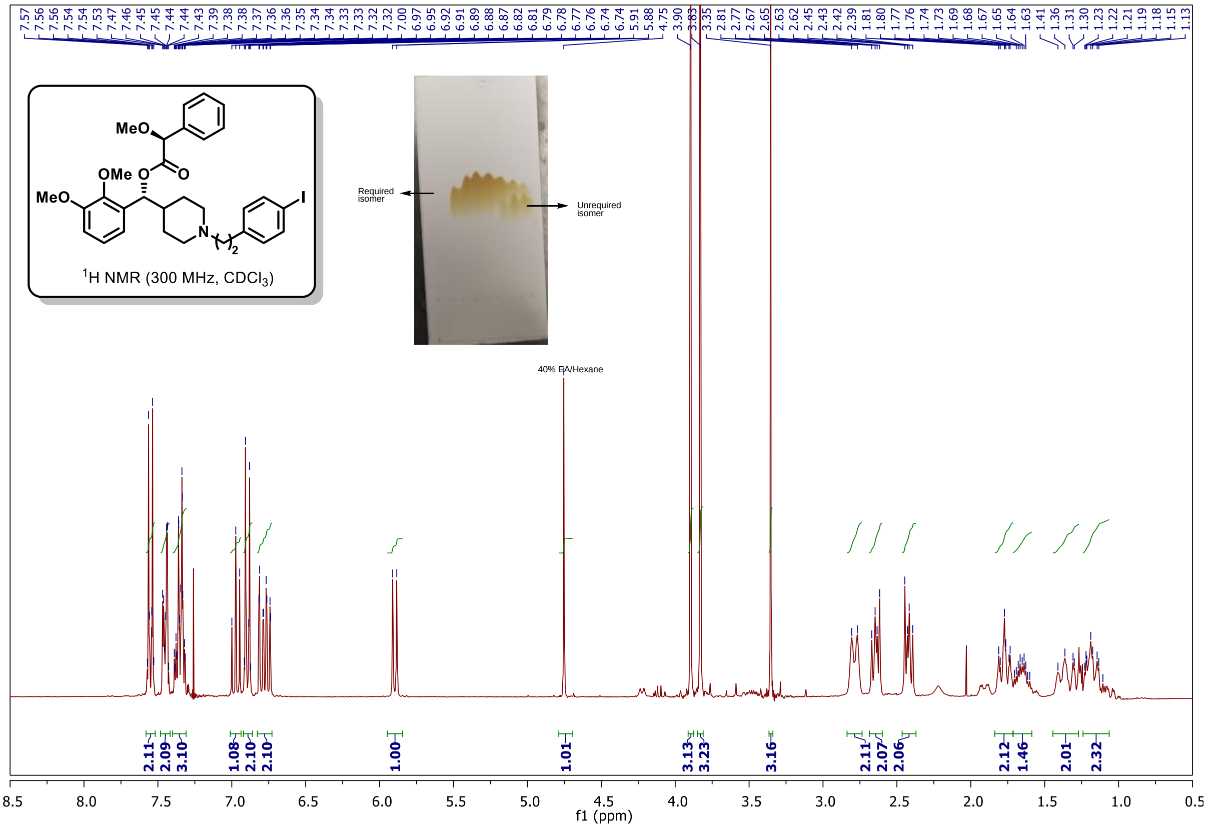

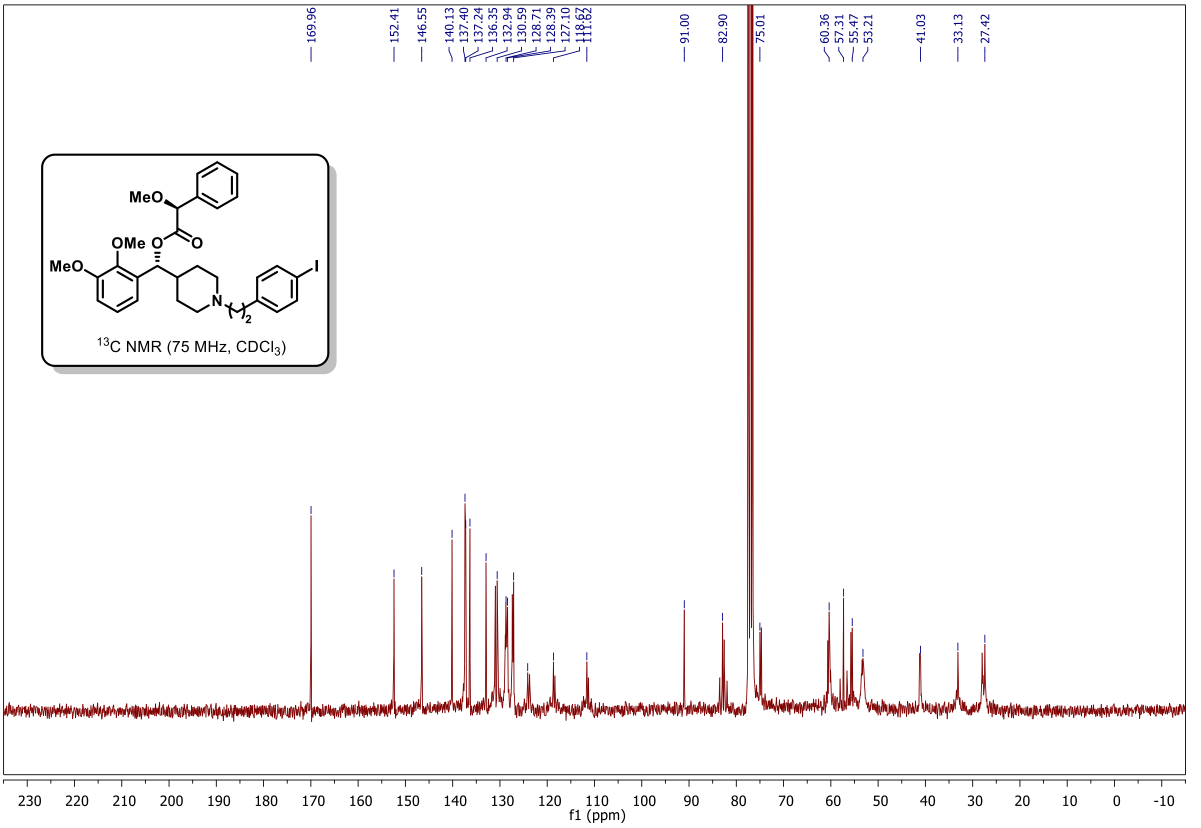

Figure S10. ^{1}HNMR (top) and ^{13}CNMR (bottom) of (*R*)-(2, 3-dimethoxyphenyl)(1-(4-iodophenethyl)piperidin-4-yl)methyl 2-methoxy-2-phenylacetate **(14a)**.

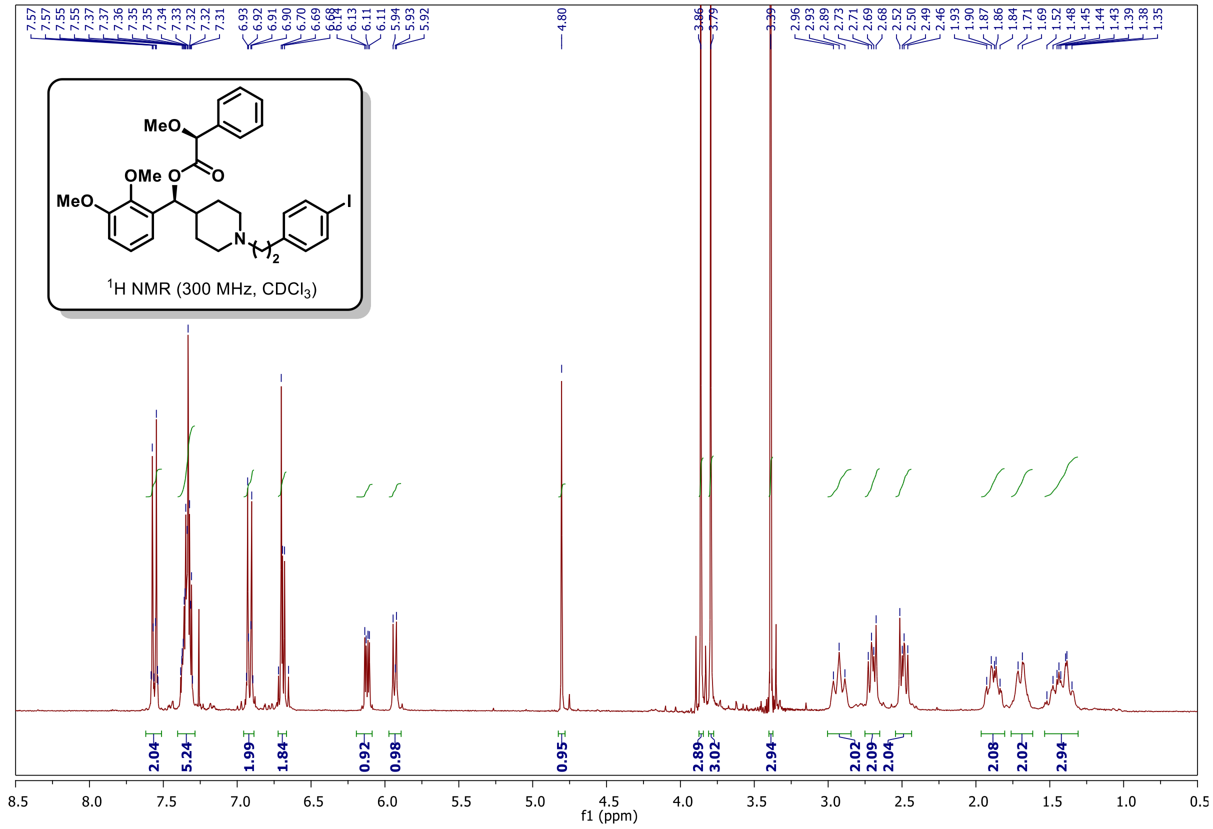

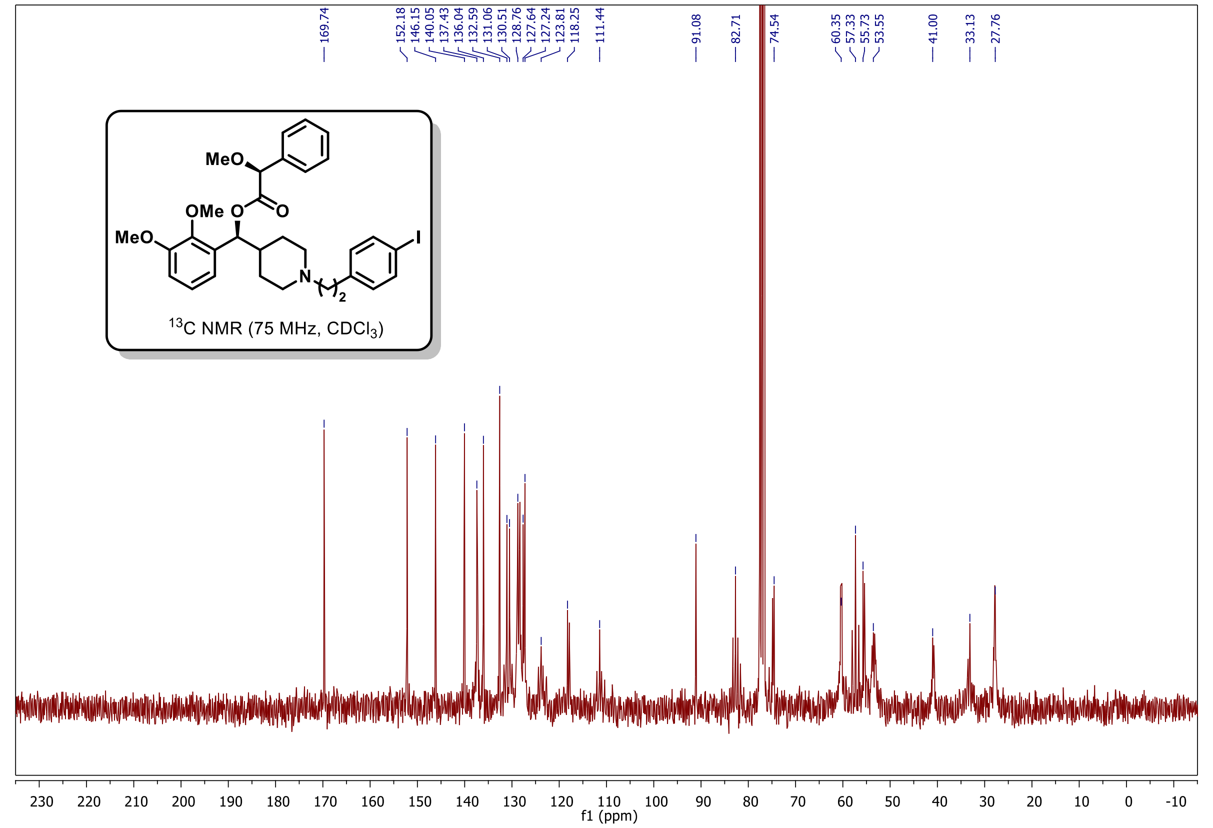

Figure S11. ^{1}HNMR (top) and ^{13}CNMR (bottom) of (*S*)-(2, 3-dimethoxyphenyl)(1-(4-iodophenethyl)piperidin-4-yl)methyl 2-methoxy-2-phenylacetate **(14b)**.

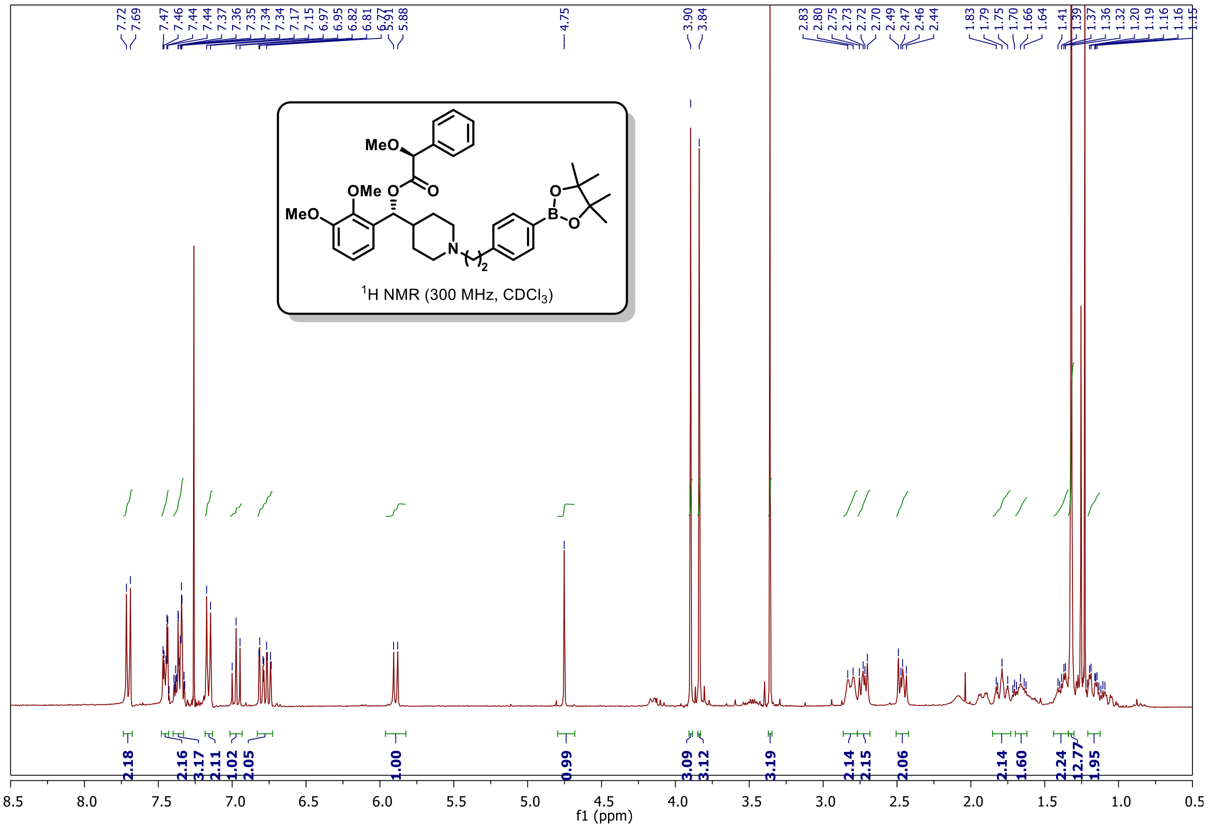

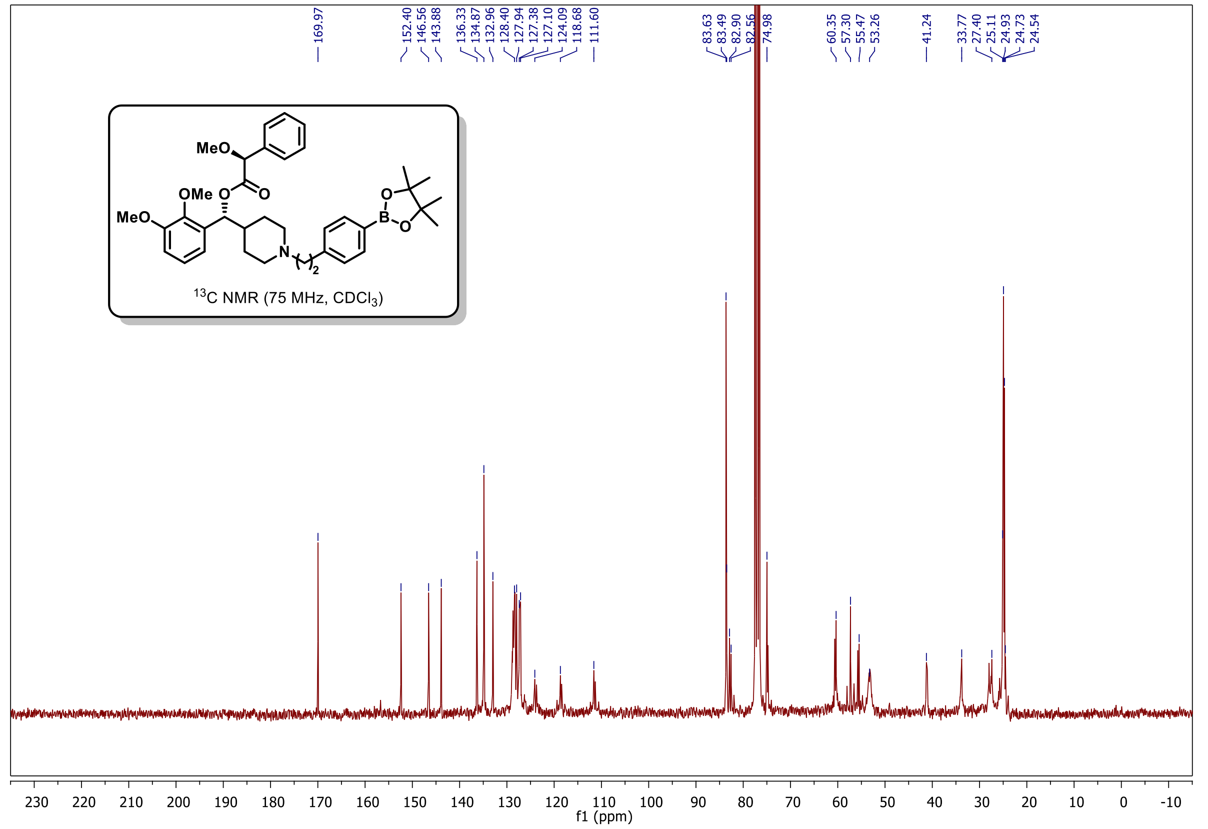

Figure S12. ^{1}HNMR (top) and ^{13}CNMR (bottom) of (*R*)-(2, 3-dimethoxyphenyl)(1-(4-(4,4,5,5-tetramethyl-1,3,2-dioxaborolan-2 yl)phenethyl) piperidin -4-yl)methyl (*S*)-2-methoxy-2-phenylacetate **(15)**.

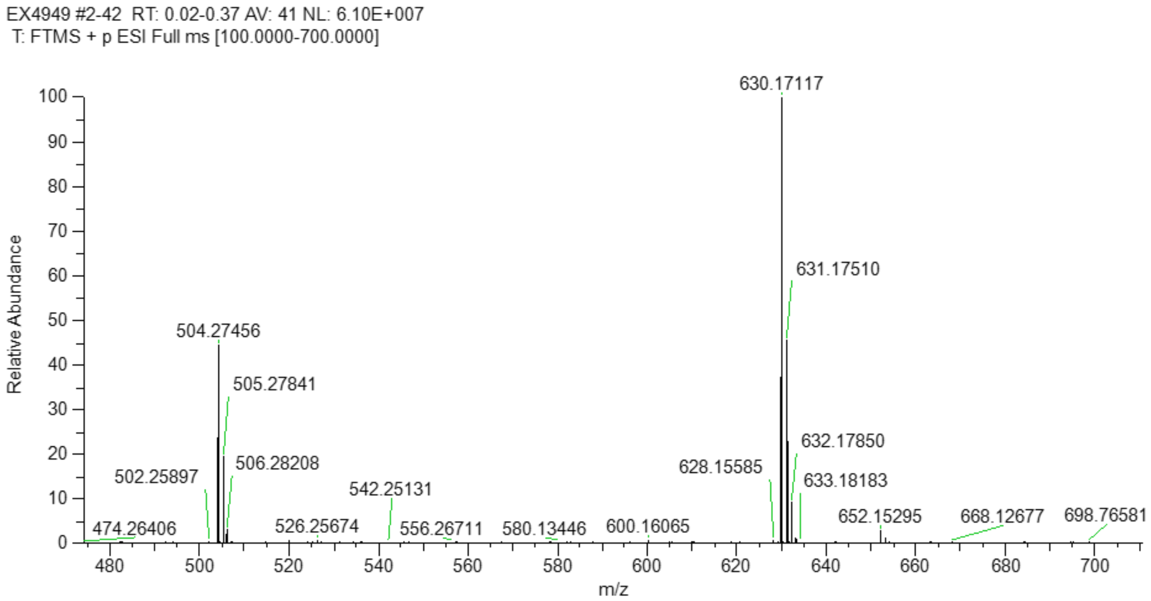

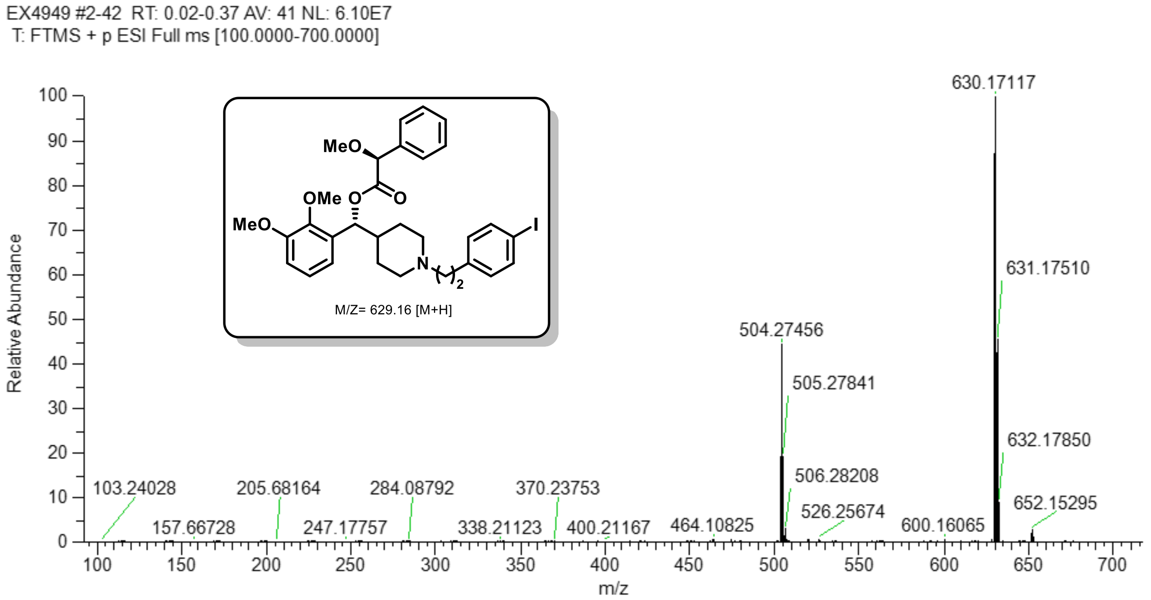

Figure S13. HRMS of (*R*)-(2, 3-dimethoxyphenyl)(1-(4-iodophenethyl)piperidin-4-yl)methyl 2-methoxy-2-phenylacetate **(14a)**.

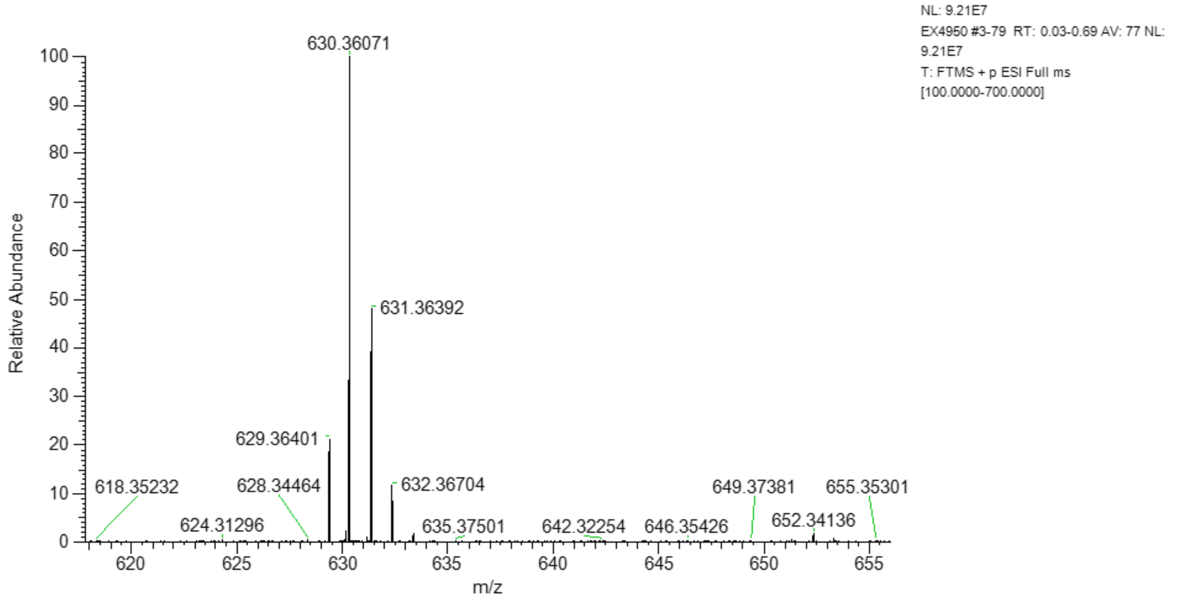

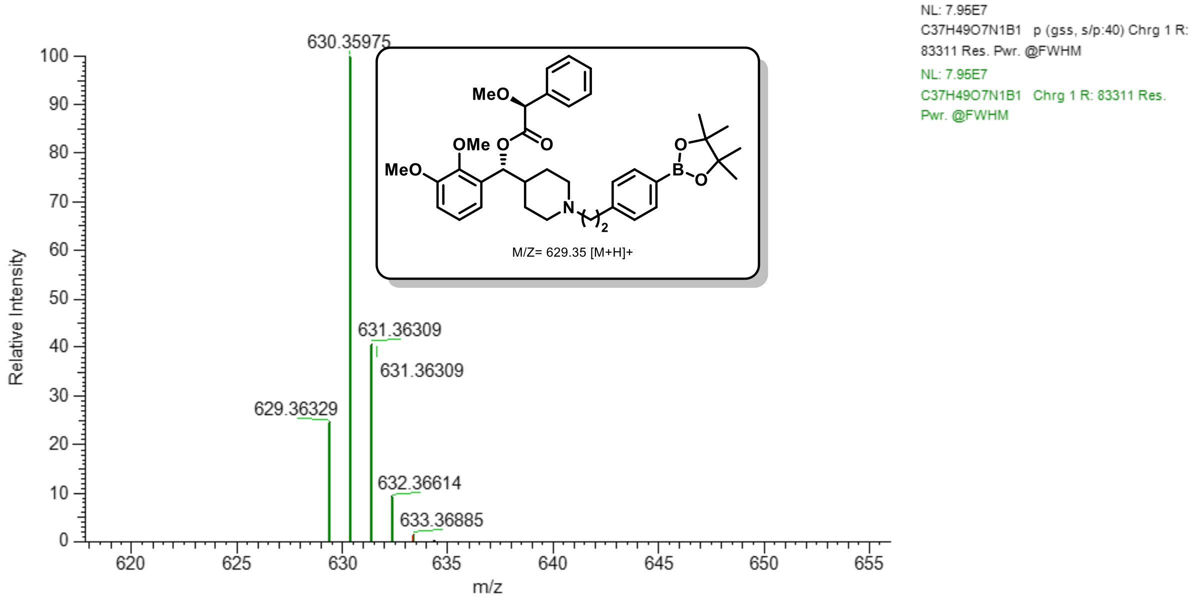

Figure S14. HRMS of (*R*)-(2, 3-dimethoxyphenyl)(1-(4-(4,4,5,5-tetramethyl-1,3,2-dioxaborolan-2 yl)phenethyl) piperidin -4-yl)methyl (*S*)-2-methoxy-2-phenylacetate **(15)**.

- **Prep HPLC method:**
  Semi prep column: (waters X Terra Prep RP18, 5 um, 19 X 100 mm column)
  Solvent System: 50%, H_2O/EtOH, 0.1% TFA
  Solvent flow rate: 6 mL/min
  Detectors: Radioactive probe, product collected at ~15.5 min

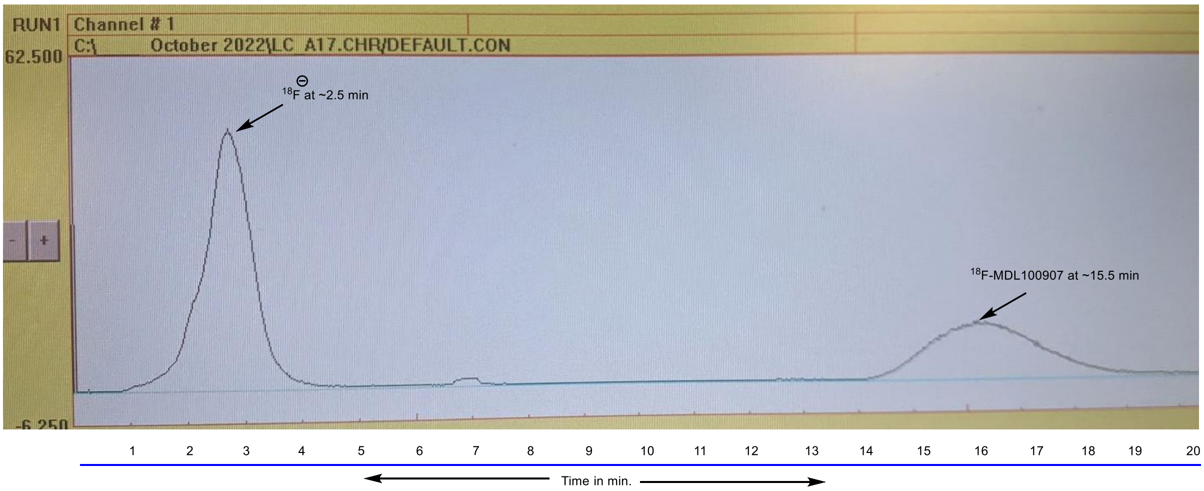

Figure S15. Prep HPLC radioactivity-probe detector scan: of (+)-[^{18}F]- MLD100907(**11)** HPLC purification.

- **Analytical HPLC method:**
  Solvent System: 50%, H_2O/EtOH, 0.1% TEA
  Solvent flow rate: 2 mL/min, product observed at ~8 min
  Analytical prep column: (waters X Terra Prep RP18, 5 um, 7.8 X 100 mm column)

Waters in WW 241

Project Name: Carbo___Metabolite

Reported by User: System

SAMPLE INFORMATION

Sample Name:	M100, Crude + cold M100	Acquired By:	System
Sample Type:	Unknown	Sample Set Name:	
Vial:	6	Acq. Method:	_ S 220 254 Varied @ 2
Injection #:	1	Date Acquired:	10/17/2022 1:22:49 PM
Run Time:	20.00 Minutes	Injection Volume:	10.00 ul

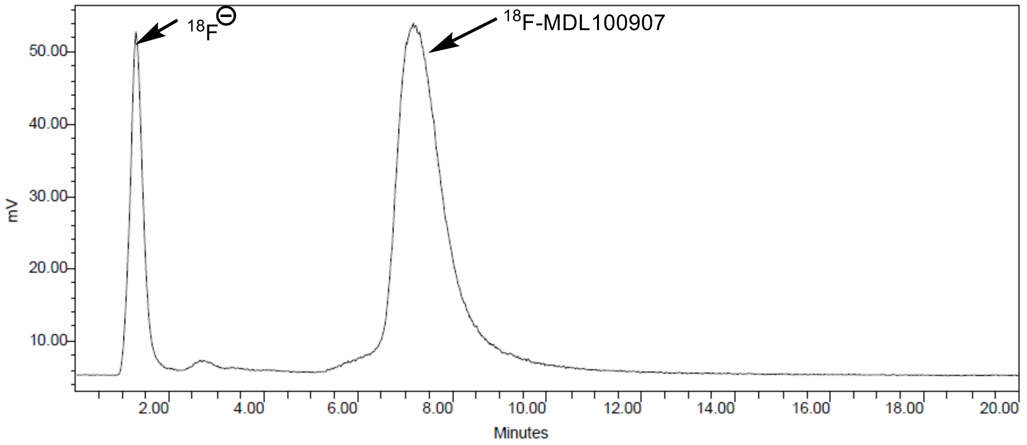

Figure S16. Analytical HPLC radioactivity detector scan: of (+)-[^{18}F]- MLD100907(**11)** crude reaction mixture.

Waters in WW 241

Project Name: Carbo___Metabolite
Reported by User: System

SAMPLE INFORMATION

Sample Name:	M100, Crude + cold M100	Acquired By:	System
Sample Type:	Unknown	Sample Set Name:	
Vial:	6	Acq. Method:	_ S 220 254 Varied @ 2
Injection #:	1	Date Acquired:	10/17/2022 1:22:49 PM
Run Time:	20.00 Minutes	Injection Volume:	10.00 ul

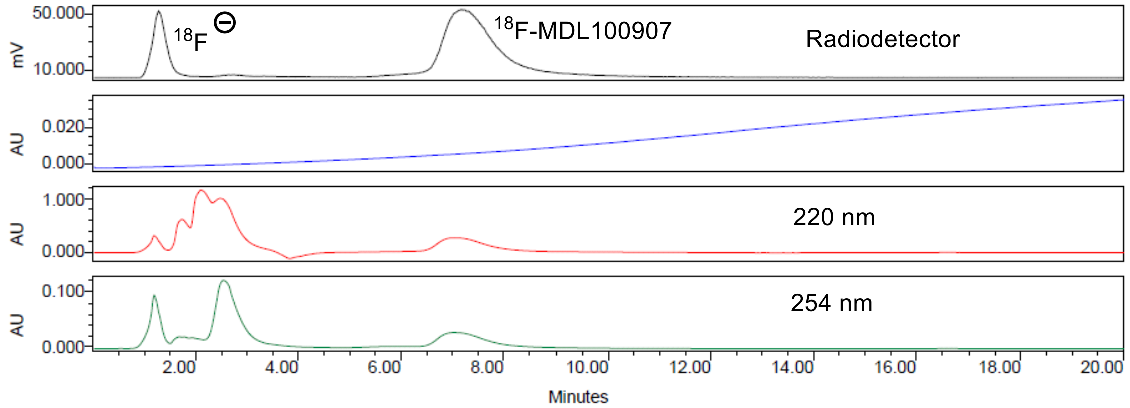

Figure S17. Analytical HPLC radioactivity detector and UV 220 nM and 254 nM detector scan: of (+)-[^{18}F]- MLD100907 **(11)** crude reaction mixture.

CSI @ Emory

Project Name: WW_248_2014
Reported by User: System

SAMPLE INFORMATION

Sample Name:	M100907, Dose	Acquired By:	System
Sample Type:	Unknown	Sample Set Name:	
Vial:	2	Acq. Method:	_S 254 280 @ 2
Injection #:	1	Date Acquired:	10/10/2022 11:23:11 AM
Run Time:	15.00 Minutes	Injection Volume:	10.00 ul

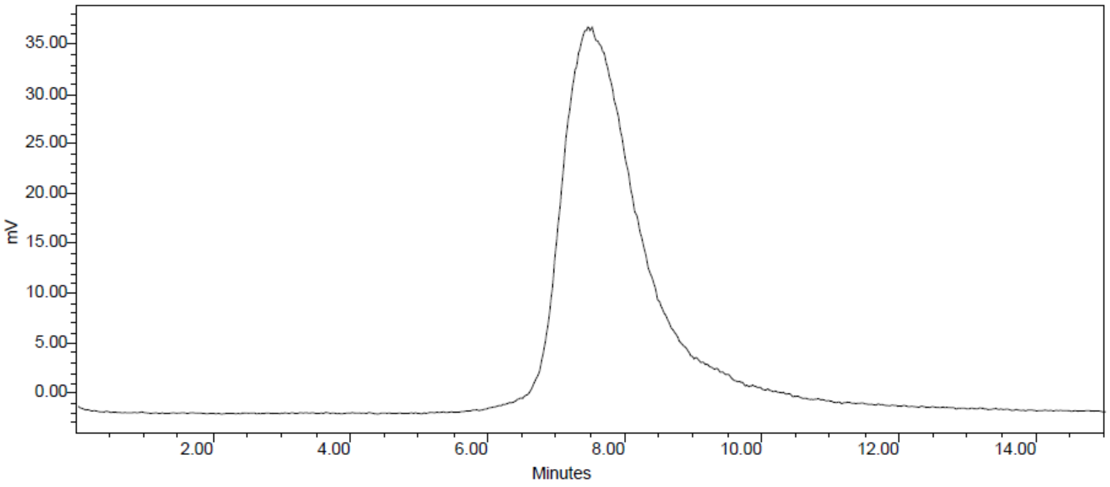

Figure S18. Analytical HPLC radioactivity detector scan: of (+)-[^{18}F]- MLD100907 **(11)** purified dose.

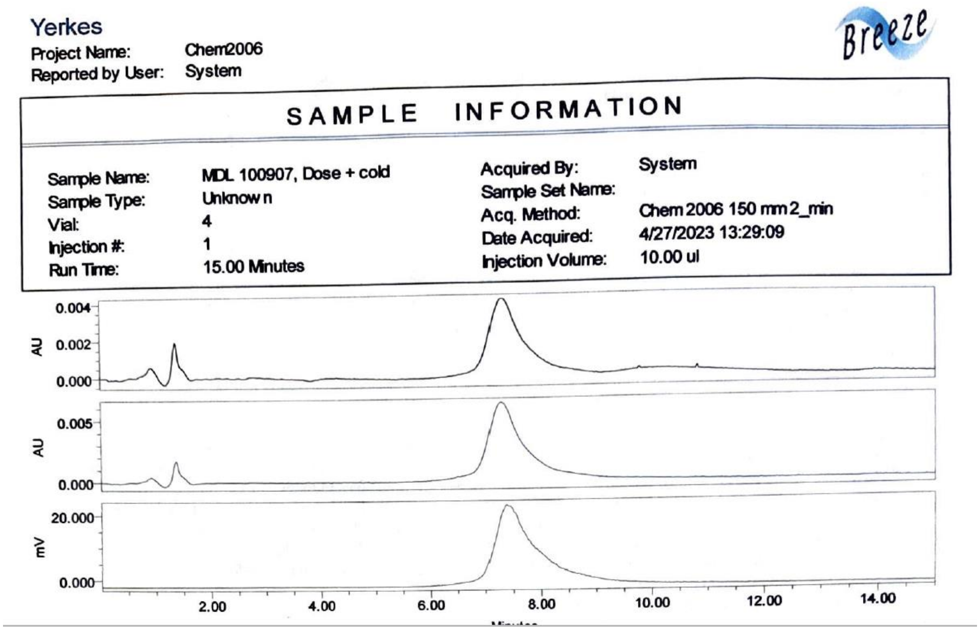

Figure S19 Analytical HPLC radioactivity detector and UV 254 nM and 280 nM detector scan: of (+)- [^{18}F]- MLD100907 **(11)** and (+)- [^{19}F]- MLD100907 **(11)**.

- **Chiral HPLC method:**
  Chiral column: (Phenomenex Lux@ 5 um Cellulose-1, LC 250 X 4.6 mm column)
  Solvent System: Hexane/Isopropanol/DIPEA, 95:5:02
  Solvent flow rate: 0.5 mL/min
  Detectors: Radioactive probe, product observed at 52.1 min
- **^{18}F-MDL100907 in EtOH:**

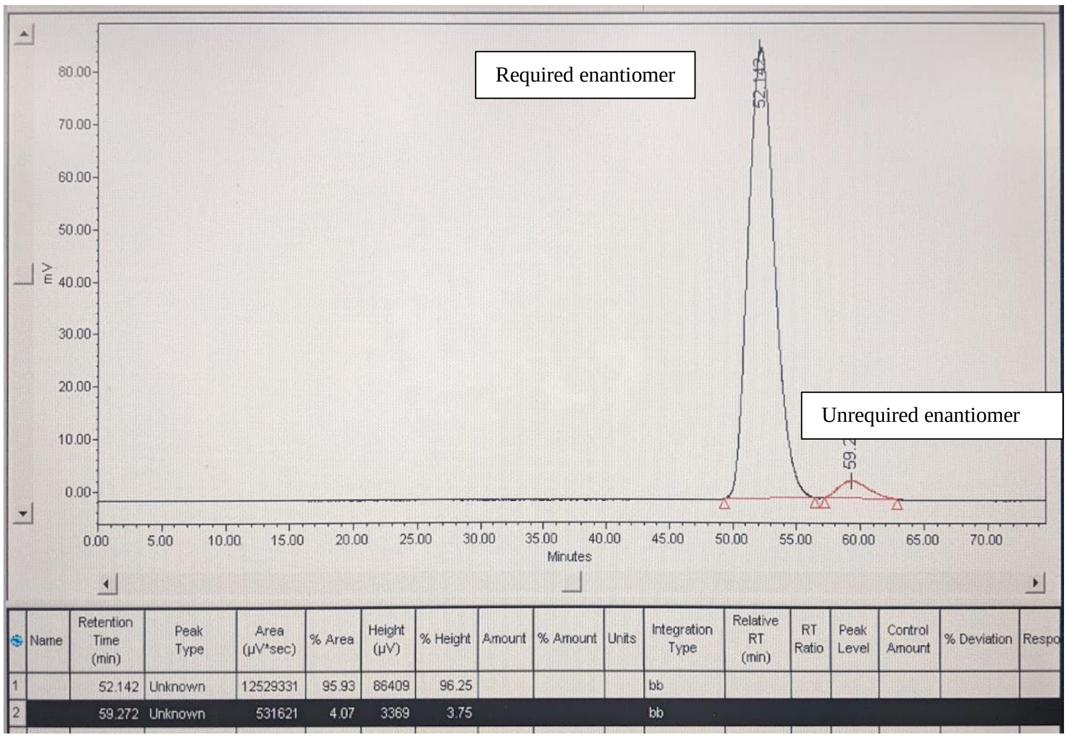

	Name	Retention Time (min)	Peak Type	Area (µV*sec)	% Area	Height (µV)	% Height	Amount	% Amount	Units	Integration Type	Relative RT (min)	RT Ratio	Peak Level	Control Amount	% Deviation	Respo
1		52.142	Unknown	12529331	95.93	86409	96.25				bb						
2		59.272	Unknown	531621	4.07	3369	3.75				bb						

Figure S20. Analytical Chiral HPLC radioactivity detector scan: of [^{19}F]- MLD100907 **(11)**.

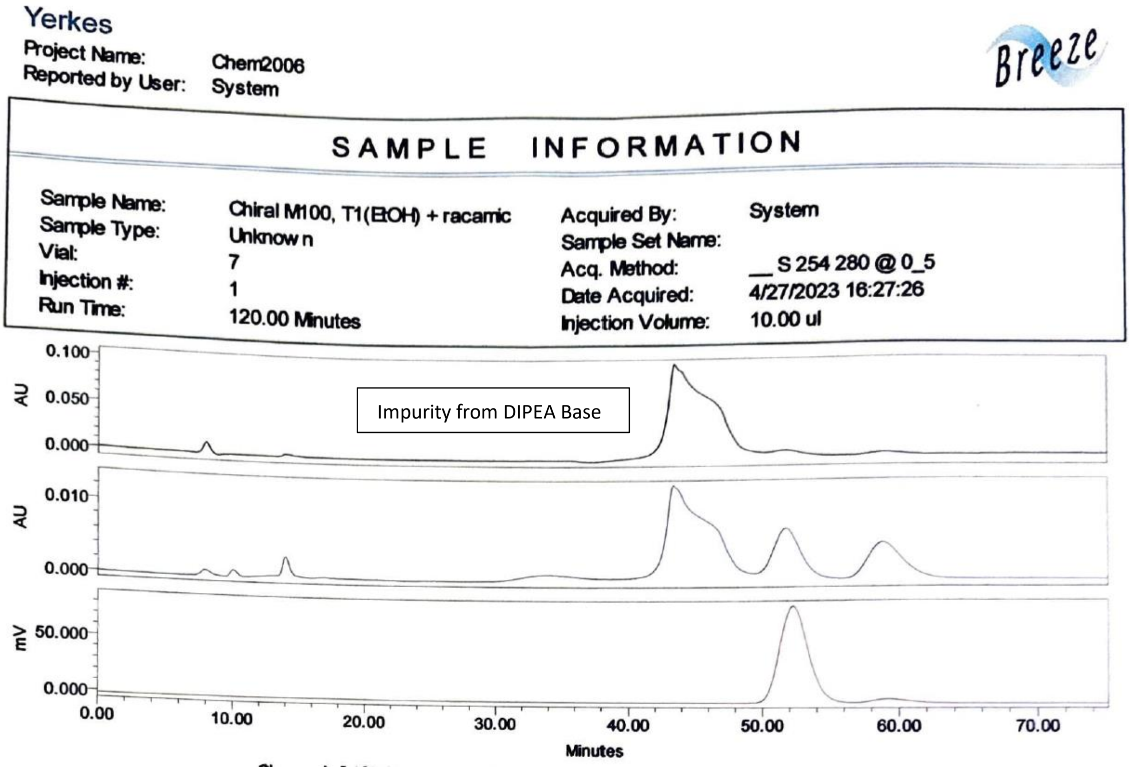

Figure S21. Analytical Chiral HPLC radioactivity and UV 280 nM detector scan comparison of (+)-[18F]- MLD100907 **(11)** dose with racemic 19F-MDL100907.

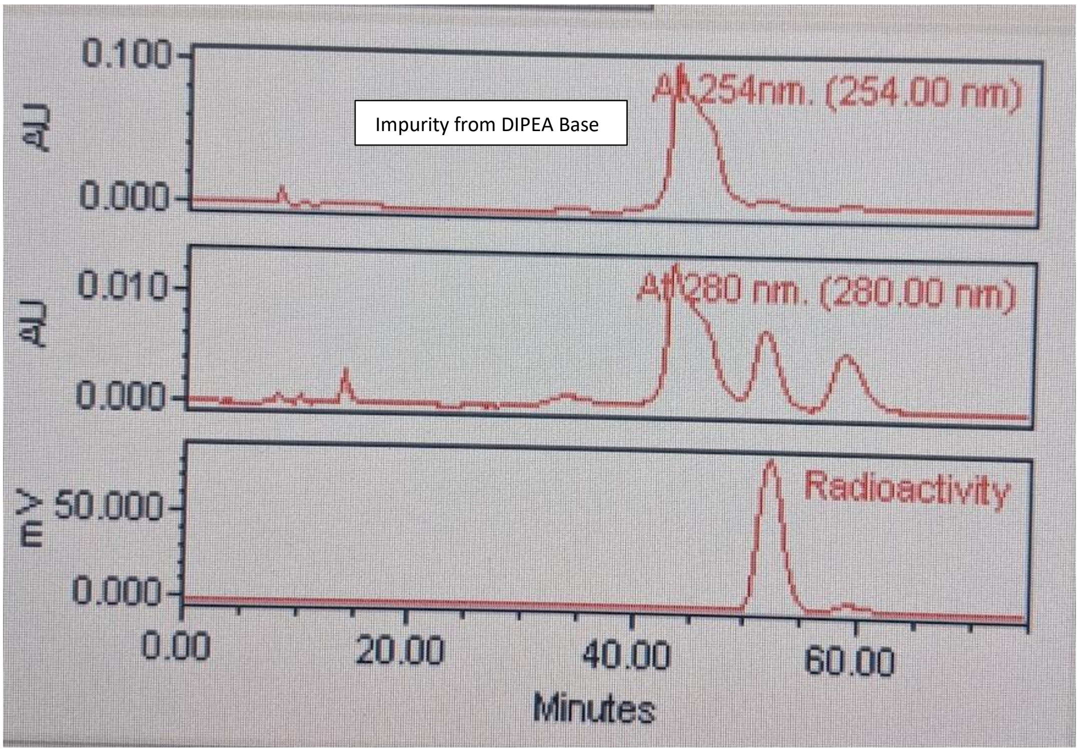

Figure 22. Analytical Chiral HPLC radioactivity and UV detectors at 254nM and 280 nM scan comparison of [18F]- MLD100907 **(11)** dose with racemic 19F-MDL100907.

Table S1. ^{18}F-MDL100907 production runs AVG DCRCY +/-SD:

Date	Total mCi Product	Starting mCi DC-Total Product	%DC-RCY
8/18/2022	198	395	50
8/29/2022	90	399	23
9/12/2022	150	477	31
9/19/2022	147	390	37
10/03/2022	121	414	29
10/10/2022	78	402	19
10/17/2022	147	432	34
AVG DCRCY +/-SD			32 +/- 10

- **Determination of molar activity of ^{18}F-MDL100907:**

The amount of non-radioactive carrier was determined from the peak area in UV–HPLC chromatograms using an UV absorbance/concentration calibration curve (λ = 280 nm). Solution of different volumes of the concentration were injected on the HPLC. The solutions of radiolabeled products obtained after HPLC purification remained at ambient temperature for at least 24 h. 50 µL of the resulting dose solution was injected into the HPLC system (50 µL loop, equals 10% of total carrier content). The peak area was determined (Multokrom- 100, 250×4.6 mm column; 50% EtOH/H_2O, 0.1% triethylamine), and the amount of carrier was calculated according to the calibration curve.

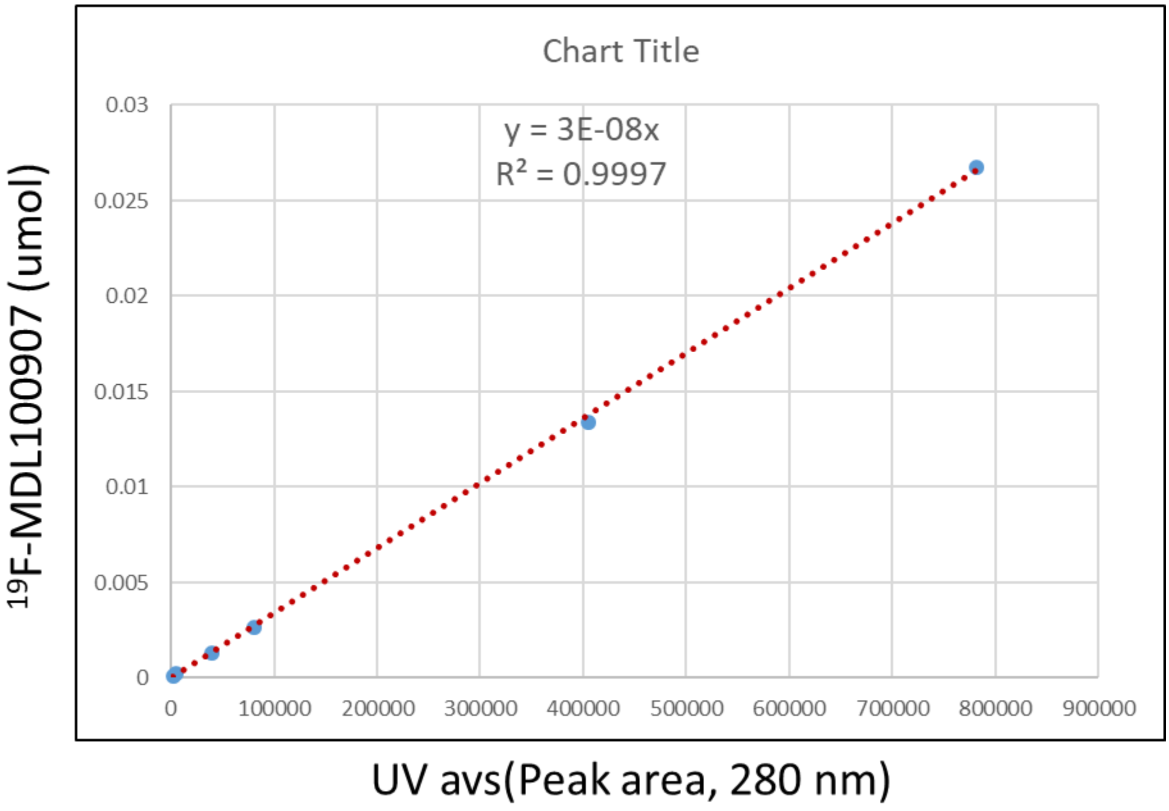

Figure S23. Standard curve of amount vs UV absorbance of ^{19}F-MDL100907
Three of 50 µL of ^{18}F-MDL100907 (10.64 mCi/mL @EOB), decayed overnight, samples were injected to the analytical HPLC. The average UV absorbance was 24539 (±5%), corresponding to 2.4 nmol of ^{19}F-MDL100907. The molar activity of ^{18}F-MDL100907 was calculated to be 2.1 Ci/µmol @ EOB.

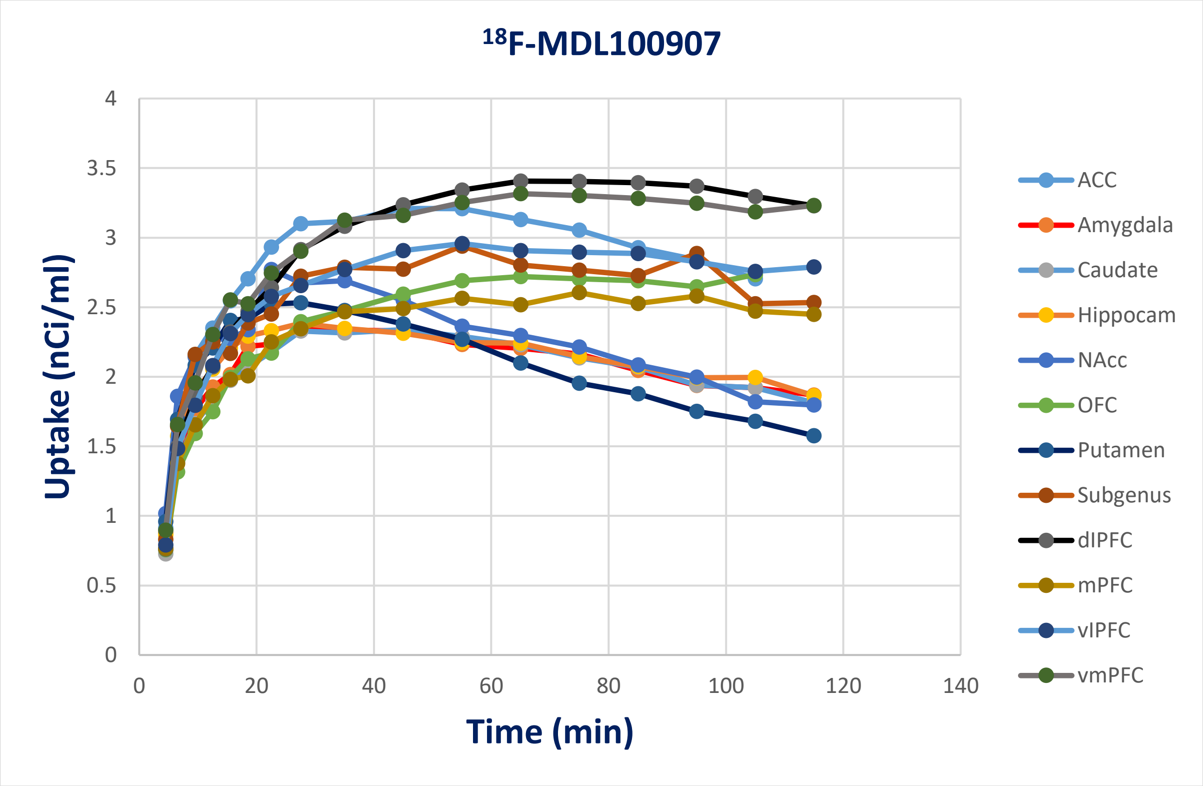

Figure S24. Time activity curve (TAC) of (+)-[18F]-MDL100907 in a male rhesus monkey for 0-120 min post injection.

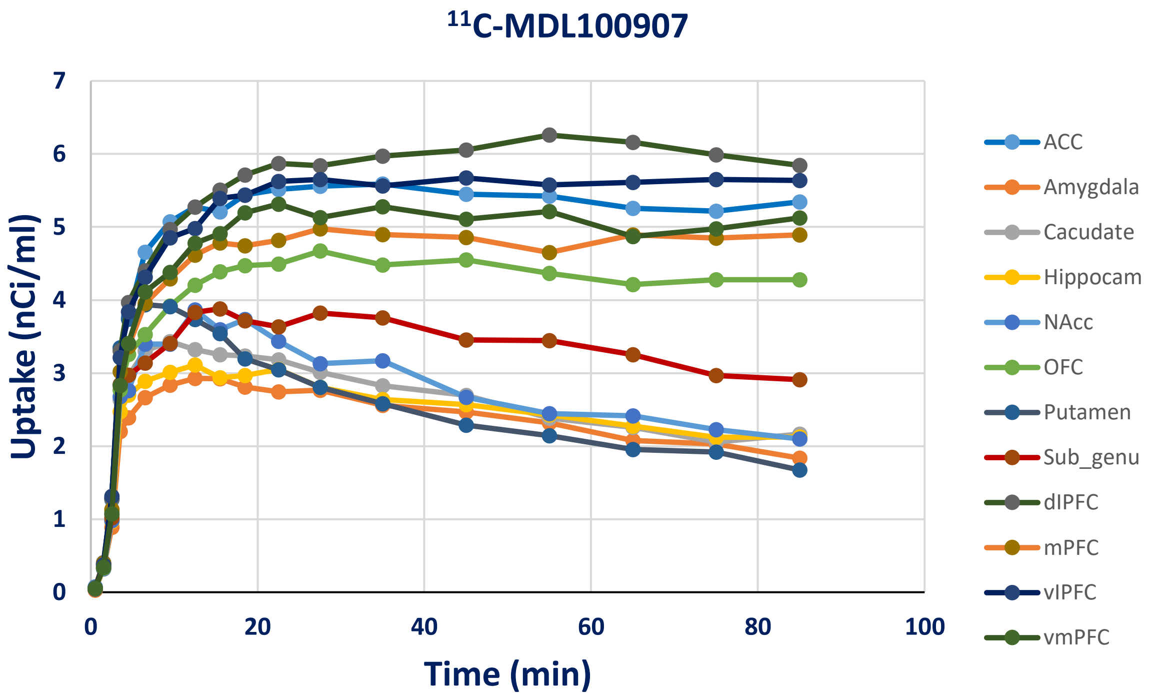

Figure S25. Time activity curve (TAC) of (+)-[11C]-MDL100907 in the same male rhesus monkey 3 years earlier for 0-90 min post injection.
